# Fitness Landscapes of APOBEC3G Antagonism by HIV-1 Vif proteins

**DOI:** 10.1101/2025.10.20.683452

**Published:** 2025-10-20

**Authors:** Caroline A. Langley, Michelle Lilly, Harmit S. Malik, Michael Emerman

**Affiliations:** 1 Molecular and Cellular Biology Graduate Program, University of Washington, Seattle, WA; 2 Division of Human Biology, Fred Hutchinson Cancer Center, Seattle, WA; 3 Division of Basic Science, Fred Hutchinson Cancer Center, Seattle, WA; 6 Howard Hughes Medical Institute, Fred Hutchinson Cancer Center, Seattle, WA

## Abstract

Host immune factors shape viral evolution. The HIV-1 Vif protein counteracts viral hypermutation caused by the host cytidine deaminase APOBEC3G (A3G), ensuring productive infection. Using deep mutational scanning (DMS) across two divergent HIV-1 clade B Vif proteins, we systematically mapped the mutational landscape governing their antagonism of A3G. These high-resolution fitness maps define conserved and adaptable regions of Vif, illuminating core principles of host–virus coevolution. Most missense mutations were strongly deleterious, reflecting pervasive purifying selection. Yet several highly conserved residues at binding interfaces with A3G, RNA, and CBFβ exhibited unexpected mutational tolerance, revealing structural flexibility at these sites. Comparative analysis revealed shared constraints and striking differences between HIV-1 strains, shaped by epistatic interactions and additional selective pressures, including Vif antagonism of A3H and PP2A. By pinpointing evolutionary vulnerabilities and adaptive mechanisms, this study provides a framework for understanding viral plasticity and developing targeted strategies to disrupt Vif-mediated immune evasion.

## Introduction

The interplay between viral proteins and host restriction factors drives a dynamic evolutionary arms race, imposing intense selective pressures on both viruses and their hosts (1–8). Host species evolve mechanisms to recognize and neutralize viral components, whereas viruses evolve countermeasures to subvert these defenses. This reciprocal adaptation fosters continuous cycles of molecular innovation that enhance viral persistence and transmission, while simultaneously shaping host antiviral responses over evolutionary timescales. Such host-virus conflicts have profoundly influenced the evolutionary trajectories of lentiviruses, including Human Immunodeficiency Virus type 1 (HIV-1) and its Simian Immunodeficiency Virus (SIV) precursors in non-human primate species.

Among the most potent innate defenses against HIV-1 are encoded by members of the Apolipoprotein B mRNA Editing Enzyme Catalytic Polypeptide-like 3 (APOBEC3, or A3) family of cytidine deaminases. In primates, this gene family comprises seven enzymes (A3A through A3H), with A3G being the most effective inhibitor of HIV-1 (9). The antiviral activity of A3G depends on its incorporation into budding virions and subsequent delivery to target cells, where it localizes to the site of viral reverse transcription. Upon encountering single-stranded viral DNA, A3G deaminates cytidine residues at the second position of 5′-CC dinucleotides (10, 11), resulting in G-to-A hypermutation in the nascent HIV-1 reverse transcripts (10–13). These mutations often introduce missense or nonsense changes into the viral genome, severely impairing replication and blocking productive infection. To counteract A3G and other A3 family members, HIV-1 encodes the viral infectivity factor (Vif), a multifunctional protein that prevents A3G incorporation into virions by promoting A3G degradation within the producer cell, thereby neutralizing its antiviral effects (14, 15). This function is mediated through Vif’s recruitment of several host cofactors: Elongin B (EloB), Elongin C (EloC), Cullin 5 (Cul5), and core-binding factor β (CBFβ). Together, these host factors form an E3 ubiquitin ligase complex known as the VCBC complex that ubiquitinates A3G, targeting it for proteasomal degradation and reducing the risk of deleterious hypermutation in the viral genome (14–20).

Molecular differences in A3 proteins between hominids and Old-World monkeys have posed significant barriers to the cross-species transmission of lentiviruses, including SIVs and HIV-1 (21, 22). These interspecies differences have driven the evolutionary diversification of Vif, selecting for mutations that enable antagonism of the distinct A3 proteins found in different primate hosts (23–25). Comparative analyses of primate A3G proteins and lentiviral Vif sequences have pinpointed key interaction surfaces and adaptive substitutions at the Vif-A3G interface (24–26). One notable example of such adaptation is the emergence of a histidine residue at position 83 in Vif, which arose during the transmission of a recombinant SIV to chimpanzees. This 83H substitution enabled SIV_cpz_ Vif (in chimpanzees) to counteract hominid A3G, thereby overcoming a critical species barrier and enabling the eventual emergence of HIV-1 in humans (25).

Structural and evolutionary analyses have significantly advanced our understanding of Vif-A3G antagonism. Recent high-resolution cryo-EM structures of HIV-1 Vif bound to human A3G have corroborated and expanded upon comparative evolutionary studies, validating key A3G-interacting residues and uncovering additional contacts, particularly RNA-binding residues that stabilize the Vif-A3G interface through an RNA-mediated scaffolding mechanism (26–28). Vif sequences from individuals living with HIV-1 exhibit extensive natural variation, including numerous polymorphisms that modulate the antagonism of A3 proteins or other host factors such as PP2A (29–42). A recent comprehensive analysis of global HIV-1 *vif* diversity identified sites evolving under either purifying selection or pervasive diversifying selection (29), underscoring both ongoing constraints and adaptive pressures acting on this gene. This persistent sequence variability raises critical unanswered questions about Vif’s mutational landscape, its adaptive flexibility, and how natural variation impacts viral evolution, immune evasion, and pathogenesis.

While comparative sequence analyses can provide important insights, they reflect the combined impact of all pressures acting on Vif: multiple restriction factors, cofactors, immune responses, genomic constraints such as overlapping reading frames, as well as non-selective founder effects. As a result, evolutionary conservation alone cannot disentangle which constraints are specific to the Vif-A3G arms race. For instance, residues that appear invariant across isolates may be mutationally flexible with respect to A3G antagonism but conserved because they are required for antagonizing other host proteins or because of overlap with essential viral genes such as *pol*. Conversely, residues that would otherwise be constrained to maintain A3G antagonism may evolve rapidly if shaped by competing interactions. Thus, while conservation integrates the totality of selective pressures on Vif, it obscures the specific contributions of A3G.

Deep mutational scanning (DMS), a powerful high-throughput approach that enables systematic evaluation of the functional consequences of nearly all possible single missense substitutions within a protein (43–45), offers a complementary experimental lens. By subjecting large variant libraries to defined selection pressures in pooled assays, DMS directly reveals how individual mutations influence protein stability, intermolecular interactions, and overall fitness. Initially developed to interrogate the functional landscapes of enzymes and essential housekeeping genes (43–45), DMS has since been applied to diverse biological systems, offering critical insights into host-virus interactions and mechanisms of viral escape from innate and adaptive immunity (46–50). Applied to Vif, DMS allows us to deconvolve the constraints imposed by A3G from the other evolutionary forces that conservation reflects.

Here, we applied DMS to systematically investigate the *vif* genes of two HIV-1 Clade B strains. The first is the LAI reference strain, which was used in the cryo-EM structure of the Vif-A3G complex (26). The second is the 1203 strain, a primary clinical isolate derived from an individual homozygous for A3H haplotype II, which encodes the most potent antiviral variant of this gene. Consequently, its Vif evolved under dual selective pressure to counter both A3G and A3H restriction factors (51, 52). Given that A3G is the most potent anti-HIV-1 restriction factor (9, 53), we aimed to define the structural and functional constraints on Vif evolution shaped exclusively by this pressure and compare how evolutionary context shapes adaptive potential. We observed a strong inverse correlation between A3G-mediated G-to-A hypermutation and variant enrichment, confirming that our DMS-derived fitness scores reliably capture Vif’s ability to neutralize A3G. Across both strains, most missense mutations reduced viral fitness, reflecting strong purifying selection. This constraint was particularly pronounced at known interfaces with A3G and CBFβ, consistent with their critical functional roles. Nonetheless, we identified pockets of mutational tolerance, even at residues that are perfectly conserved across HIV-1 and SIV_cpz_. This includes residue 42, which binds RNA, and site 83 within the Vif-A3G “arms-race” interface, highlighting areas of unexpected adaptive flexibility in A3G antagonism (26). Comparative analysis revealed 56 missense variants across 38 positions that have significantly divergent fitness consequences between LAI and 1203 Vif, illustrating how strain-specific selective pressures have divergently shaped the mutational landscape of Vif. Together, these findings offer a comprehensive, high-resolution map of Vif’s evolutionary plasticity, illuminating the trade-offs between maintaining essential antiviral countermeasures and exploring alternative adaptive solutions in the ongoing host–virus arms race.

## Results

### A pooled functional selection assay for HIV-1 *vif* variants

**Figure 1:**
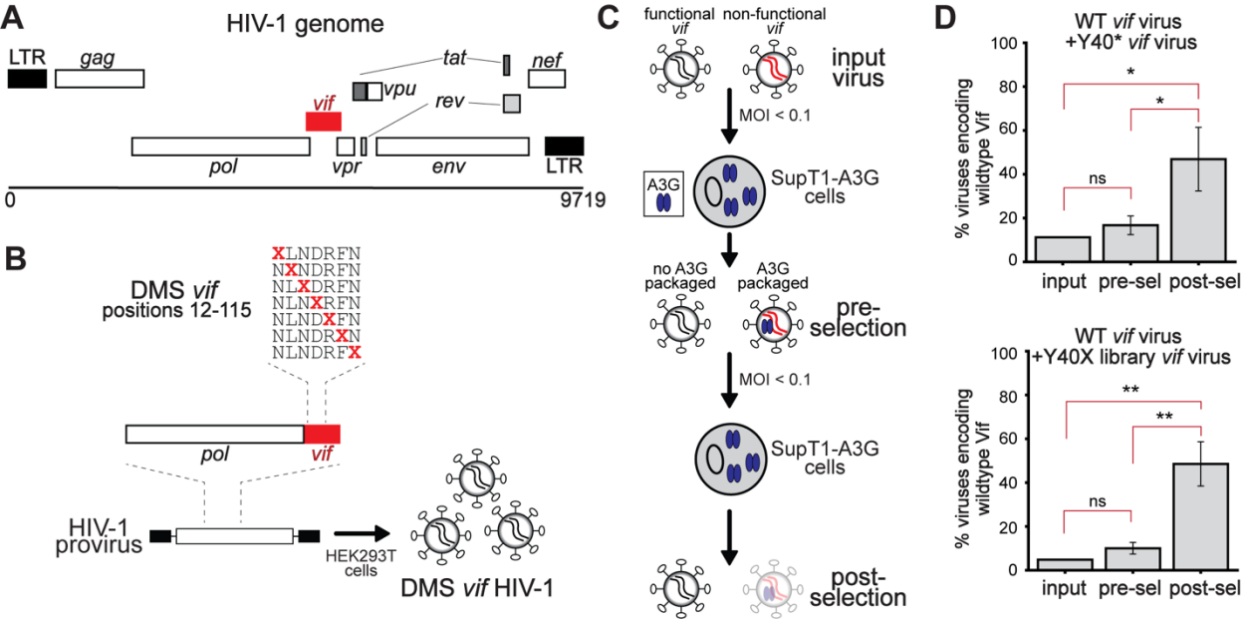
Selection of functional Vif variants through passaging in A3G-expressing cells. A. Schematic of the HIV-1 genome with the *vif* gene highlighted in red. The 5’ end of *vif* overlaps with the Integrase coding region in *pol*. B. Codon-specific DMS libraries were generated across amino acid positions 12–115 of Vif from two HIV-1 clade B strains (LAI and 1203). A replication-competent HIV-1 proviral backbone was used in which the endogenous *vif* start codon was repositioned downstream of the pol reading frame to eliminate overlap, as described previously (21). DMS Libraries were cloned into this backbone and transfected into HEK293T cells to produce infectious DMS virus pools. C. DMS virus pools were used to infect SupT1 cells stably expressing physiological levels of A3G (SupT1-A3G) at a low multiplicity of infection (MOI < 0.1). Viruses encoding functional Vif (gray) replicate efficiently, whereas those encoding non-functional Vif (red) incorporate A3G (blue ovals) into virions and are depleted upon subsequent infection. Viral supernatant was harvested 72 hours after the first infection (“pre-selection”) and used to infect fresh SupT1-A3G cells. During the second passage, packaged A3G (blue ovals) deaminates cytidines on minus-strand cDNA, introducing mutations. Seventy-two hours later, “post-selection” supernatant was harvested for deep sequencing of viral RNA to quantify enrichment or depletion of individual Vif variants. D. Validation of the selection assay using control virus mixtures. Top: Wild-type (WT) Vif virus was mixed with a premature stop codon mutant at Y40 (Y40*). Bottom: WT virus was mixed with a mini-library of substitutions at position 40 (Y40X). In both cases, the proportion of WT increased only after the second passage (“post-selection”), confirming that A3G-mediated pressure selects for functional Vif variants. Statistical significance was assessed using two-sample t-tests on biological replicates for each pairwise comparison: input vs. pre-selection, input vs. post-selection, and pre- vs. post-selection. Error bars indicate standard deviation across biological replicates. (* = p < 0.05, ** = p < 0.01, ns = not significant)

In the HIV-1 genome, the *vif* gene partially overlaps with the *pol* and *vpr* open reading frames. The first 19 codons of *vif* overlap with the 3’ end of *pol*, which encodes the C-terminus of the Integrase (IN) protein (Fig. 1A). To investigate the selective constraints governing HIV-1 Vif antagonism of A3G, we constructed comprehensive DMS libraries encompassing Vif amino acid positions 12–115 (Fig. 1B) (Supplementary data 1/2). This region was chosen based on prior studies identifying critical interfaces with A3 proteins (19, 26, 30, 54–57). We deliberately excluded the C-terminal domain (residues 116–192) because it primarily mediates interactions with the VCBC complex (17). To prevent mutations in *vif* from inadvertently disrupting IN function and confounding our readout of A3G antagonism, we removed the *pol*-*vif* overlap by introducing synonymous mutations in the *pol* reading frame and eliminating the endogenous *vif* start codon (Fig. 1B). We then repositioned the entire *vif* gene downstream of the *pol* coding region under an alternative upstream start codon, incorporating restriction sites for modular cloning into an otherwise unmodified, replication-competent HIV-1 proviral backbone, as previously described (21). This design allowed targeted mutagenesis of functionally important *vif* residues that would otherwise be constrained by genetic overlap with *pol*, including residue 15, a well-established determinant of A3G interaction (26, 58, 59).

A significant challenge with mutational analyses of Vif is that A3G can induce hypermutations in the viral genome, including within the *vif* gene itself, which could confound DMS analyses. We needed to ensure that DMS-associated substitutions could be distinguished from A3G-mediated mutations, particularly at 5′-CC dinucleotides, the preferred deamination target of A3G (10, 11). To achieve this, we eschewed conventional error-prone PCR, commonly used in DMS studies. Instead, we employed a codon-specific saturation mutagenesis strategy in which each variant contains a single, defined missense mutation traceable to a unique codon substitution at a single site, which is designed so it would not be confounded with G-to-A hypermutation events at 5′-CC dinucleotides. The resulting DMS *vif* libraries were cloned into the modified, replication-competent HIV-1 proviral backbone and transfected into HEK293T cells to generate pools of DMS *vif* HIV-1 virions (Fig. 1B).

To quantify the fitness effects of individual *vif* variants, we developed a pooled, two-passage selection assay in SupT1 cells engineered to express physiological levels of A3G (SupT1-A3G) (60) (Fig. 1C). This system provides a genetically homogenous and reproducible context, minimizing host variability and enabling precise measurement of Vif mutational effects. We used the DMS *vif* HIV-1 virions to infect SupT1-A3G cells at a low multiplicity of infection (MOI) in biological triplicate. Twenty-four hours post-infection, the viral supernatant was replaced with fresh medium to remove viral particles produced before *vif* expression. Seventy-two hours post-infection, we collected the resulting ‘pre-selection’ viral supernatant and used it to infect a fresh batch of SupT1-A3G cells (Fig. 1C). After an additional 72 hours, the ‘post-selection’ supernatant was harvested for deep sequencing (Fig. 1C).

In this system, ‘non-functional’ Vif variants that fail to counteract A3G during the initial infection are expected to lead to A3G incorporation into progeny virions, resulting in G-to-A hypermutation and reduced infectivity. These variants are depleted from the viral population in the post-selection supernatant. In contrast, ‘functional’ Vif variants that effectively restrict A3G incorporation are enriched in the post-selection supernatant relative to their pre-selection frequencies. Thus, the relative enrichment or depletion of each individual Vif variant provides a quantitative measure of viral fitness in the presence of A3G (Fig. 1C).

To validate the specificity of our assay in selecting functional *vif* variants, we performed two pilot experiments (Fig. 1D). We compared the replication dynamics of viruses encoding wild-type (WT) and a loss-of-function *vif* mutant harboring a premature stop codon at position 40 (Y40*). SupT1-A3G cells were infected with a defined mixture of WT and Y40* viruses. Given that A3G-mediated restriction becomes apparent only after two rounds of replication in A3G-expressing cells, we hypothesized that the Y40* variant would be selectively depleted in post-selection, but not pre-selection samples. To quantify this, we calculated a “selective index” as the natural logarithm of the ratio of post-selection variant frequencies to pre-selection variant frequencies: *ln(post/pre)*. Frequencies were converted to decimal form prior to transformation, such that positive values indicate enrichment and negative values indicate depletion following selection. For easier interpretability, we also computed the relative percent change as *(exp(selective index) – 1)* × *100*, reflecting the proportional change in representation. Consistent with our hypothesis, the relative abundance of WT and Y40* viruses did not differ significantly between the input and pre-selection (first passage) populations. However, in post-selection samples, the Y40* variant was markedly depleted, exhibiting selective indices of −42% and – 38% relative to the input and pre-selection populations, respectively (Fig. 1D).

In a second pilot screen, we evaluated a targeted library of missense variants at Vif position 40, a residue known to interact with RNA (26). Although this RNA interaction contributes to Vif function, it is not strictly essential, as other Vif residues also contact the same RNA nucleotide (26). This experiment enabled us to assess whether our assay could detect more nuanced selective pressures on variants with partial, rather than complete, loss of function, as seen in the Y40* mutant virus. Consistent with our expectations, we observed moderate but significant depletion of non-wild-type codons at position 40 in post-selection samples, with selective indices of −46% and −43% relative to the input and pre-selection populations, respectively (Fig. 1D). The depletion of missense variants at this position is not statistically different than the depletion of nonsense variants in our previous assay. This suggests that some missense variants could be as or even more deleterious than a truncated protein by dominantly interfering with Vif folding or RNA interactions.

The results of our pilot screens demonstrated that our assay is sensitive to both complete loss-of-function mutations and subtler impairments in Vif activity, validating the robustness of our two-passage selection system. Functional *vif* variants were consistently enriched, whereas those deficient in A3G antagonism were selectively depleted. Importantly, these effects were evident only in post-selection samples, confirming that A3G-mediated selective pressure, rather than experimental artifacts or bottlenecks, drove the observed enrichment patterns.

### Deep mutational scanning of HIV-1 LAI Vif reveals sites of mutational tolerance and constraint

We applied the validated two-passage selection assay (Fig. 1) to our complete DMS library, encompassing ~2,000 missense variants of the HIV-1 LAI Vif protein. LAI is a well-characterized, lab-adapted strain whose *vif* gene has been extensively studied (61) and was recently resolved in a high-resolution cryo-EM structure bound to A3G, RNA, and the VCBC complex (26). To quantify DMS *vif* variant fitness, we calculated log enrichment scores by comparing each variant’s frequency in post-selection replicates to its frequency in a pooled pre-selection library (merged to increase coverage and reduce sampling noise). This approach allowed the enrichment scores in each replicate to reflect the outcome of an independent selection normalized to a shared baseline. Consistency across three biological replicates was assessed using Spearman correlation coefficients between each post-selection replicate and the merged pre-selection dataset. We observed moderate to high reproducibility between replicates (LAI ρ = 0.46–0.65, Supplementary Figure 1A), consistent with previous gene-wide DMS analyses (46, 62), suggesting that replicate selections were subject to similar selective pressures.

**Figure 2:**
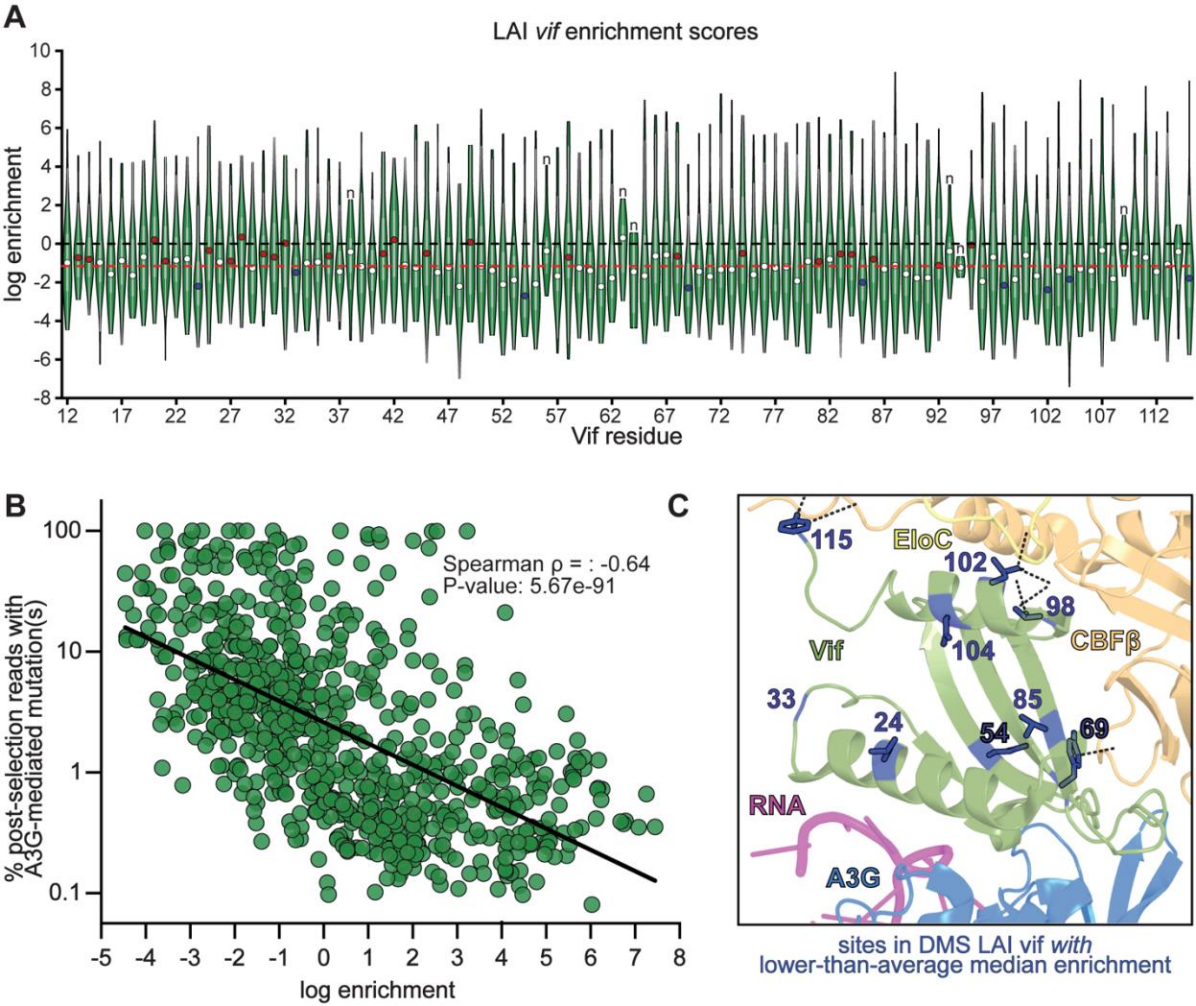
Site-level mutational tolerance of LAI Vif in the presence of A3G A. Violin plots showing the distribution of log_2_ enrichment ratios for all single missense substitutions across residues 12–115 of LAI Vif following two rounds of selection in SupT1 cells expressing A3G. Log enrichment reflects the relative fitness of each variant; higher values indicate retention or enhancement of A3G antagonism. Filled circles mark residues with significant deviation from the library-wide median: red for significantly higher and blue for significantly lower mutational tolerance. Significance was assessed using Wilcoxon signed-rank tests comparing variant enrichment values at each site to the library-wide median. Sites passing a Benjamini–Hochberg false discovery rate threshold (q ≤ 0.10) in either a two-sided test or a directionally consistent one-sided test are highlighted. Sites with insufficient variant coverage (<5 missense variants) are labeled “n”. B. Scatter plot comparing the log_2_ enrichment ratio of each variant to the percentage of post-selection reads with G-to-A mutations at predicted A3G target motifs. A strong negative correlation (Spearman ρ = −0.64, p = 5.67e–91) indicates that variants with higher fitness are more effective at suppressing A3G-induced hypermutation. C. Constrained residues (blue circles from panel A) mapped onto the cryo-EM structure of the Vif–A3G–VCBC–RNA complex (PDB: 8CX0). Bold outlines indicate sites passing the two-sided Wilcoxon test (q ≤ 0.10); thin outlines indicate sites passing the one-sided Wilcoxon test (q ≤ 0.10). Gray dashed lines denote known protein–protein interactions involving constrained residues.

To enable site-specific interpretation, enrichment scores were further normalized relative to the WT amino acid at each position, setting WT to zero so that scores reflect enrichment or depletion relative to the native residue. Median log enrichment scores across the three biological replicates were then computed for all variants at each site (Fig. 2A). This analysis revealed positions in Vif that are broadly tolerant or constrained during replication in the presence of A3G, highlighting regions important for structural integrity, cofactor interactions, and possible sites of adaptive diversification. Notably, we observed substantial variation in enrichment scores among different missense substitutions at the same position, often exceeding the differences in site-level medians. While highly enriched substitutions at several sites may reflect true adaptive potential, some extreme values likely result from assay noise due to incomplete depletion of non-functional Vif variants after one round of selection. Therefore, we focused on median scores across replicates to reduce the influence of stochastic outliers. Across the 104 targeted residues in the DMS LAI *vif* library, the overall median log enrichment score was −1.23 (Fig. 2A, red line), indicating that most single amino acid substitutions reduce viral fitness in the presence of A3G. This pattern underscores the general mutational intolerance of Vif, consistent with its critical role in counteracting host antiviral defenses.

In the absence of effective A3G neutralization, virion-incorporated A3G deaminates cytidine residues at the second position of 5′-CC dinucleotides on the minus-strand cDNA during reverse transcription (10–13). A3G’s dinucleotide preference is unique among A3 proteins (60, 63), enabling the identification of A3G-mediated G-to-A hypermutation signatures in HIV sequences derived from clinical samples (64–68). To leverage this distinctive mutational footprint as an orthogonal measure of Vif functionality, we reanalyzed the same post-selection sequencing data used for enrichment calculations. This strategy enabled us to unambiguously distinguish the encoded DMS variant from additional mutations consistent with A3G deamination within the sequenced region. We mapped known A3G target motifs in the LAI *vif* sequence and quantified the frequency of G-to-A mutations at these positions for each DMS variant. We then compared the proportion of A3G-induced mutations per variant to its log enrichment ratio (Fig. 2B). This analysis revealed a strong overall negative correlation between A3G-induced mutation burden and variant fitness (Spearman ρ = −0.64), with less-fit variants accumulating more G-to-A mutations. Variants with higher fitness (positive enrichment scores) exhibited reduced frequencies of A3G-induced mutations (mean of 4.7%), while depleted variants (negative enrichment scores) showed significantly elevated frequencies (mean of 14.5%).

To identify residues with variant effects that deviate significantly from the global trend, we tested whether the distribution of enrichment scores at each site differed from the overall protein-wide median. Specifically, we used Wilcoxon signed-rank tests to compare variant-level enrichment values at each site to the global median across all variants. Two complementary versions of the test were applied: a two-sided test to detect any deviation from the global median, and a one-sided test to capture directionally consistent enrichment or depletion. P-values from each test were corrected for multiple comparisons using the Benjamini–Hochberg procedure (69–72), applying a 10% false discovery rate (FDR) (q ≤ 0.10). Sites were considered significant if they passed the FDR threshold in either test and were reproducibly identified in at least two replicates. This dual criterion reflects the directional nature of DMS enrichment scores in Vif: sites with consistently negative enrichment values relative to the protein-wide median are inferred to be under stronger-than-average functional constraint, whereas sites with consistently positive scores are interpreted as being more mutationally tolerant than average.

Based on this criterion, we identified nine positions under significant functional constraint in the DMS LAI *vif* library (Fig. 2A, blue circles; Fig. 2C). At these sites, most substitutions were strongly depleted, suggesting that they reduce fitness relative to the WT residue. Sites 24 (73, 74), 85 (75), 102 (76), and 104 (76) fall within previously characterized motifs essential for A3G neutralization. Notably, residues 54 and 69 were the only positions that passed the more stringent two-sided Wilcoxon test (every other position only passed the one-sided test), indicating that the distribution of their enrichment scores deviated most strongly from the protein-wide median. Residues 54 and 69 were both previously implicated in A3G interaction (54, 77). Indeed, a Y69A mutation has been reported to abolish Vif-mediated antagonism of A3G (77, 78). Consistent with these findings, our DMS data showed strong depletion of the Y69A variant in the LAI *vif* library (Supplementary Table 1). Interestingly, although Y69 does not directly contact A3G in the recently resolved cryo-EM structure of the Vif–VCBC–RNA–A3G complex (26), it interacts with CBFβ (17, 26) and lies adjacent to W70, a residue critical for stabilizing the Vif-A3G interface (23–25, 59, 79). These observations suggest that Y69 contributes indirectly to A3G antagonism, likely by stabilizing local structure, orienting neighboring residues, or facilitating assembly of the VCBC complex.

**Figure 3:**
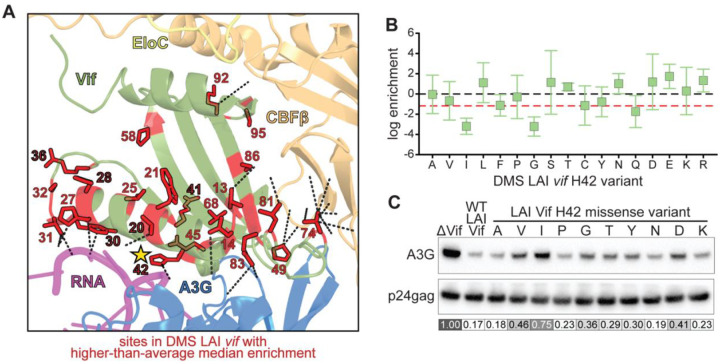
Mutational tolerance at Vif residue H42 reflects both structural flexibility and functional retention in A3G antagonism A. Sites with significantly higher-than-average median log_2_ enrichment ratios (from Fig. 2A, red circles) mapped onto the LAI Vif–A3G–VCBC–RNA cryo-EM structure (PDB: 8CX0). Sites with bold outlines passed a two-sided Wilcoxon test (BH-FDR q ≤ 0.10); sites with thin outlines passed a direction-matched one-sided Wilcoxon test (q ≤ 0.10). Gray dashed lines indicate known protein–protein interactions. Residue H42, located at the RNA-binding interface, is highlighted with a yellow star. B. Log_2_ enrichment ratios from the DMS LAI vif library for all single amino acid substitutions at position 42. The dashed black line marks the wild-type histidine (H) enrichment score; the dashed red line marks the library-wide median. C. Western blot analysis of A3G incorporation into virions following co-transfection of HEK293T cells with FLAG-tagged A3G and individual LAI Vif variants containing single substitutions at residue 42. Error bars indicate the standard deviation from three biological replicates. Numbers below each blot lane represent the relative intensity of virion-incorporated A3G, normalized to wild-type Vif.

We also identified 24 positions in the DMS LAI *vif* library with significantly higher median enrichment scores, indicative of relatively increased mutational tolerance (Fig. 2A, red circles; Fig. 3A). Although most of these sites still exhibited negative median scores, suggesting that most substitutions at these sites are deleterious, their fitness effects were notably less severe than at other regions of Vif. This pattern may reflect a weaker functional constraint or a greater degree of structural or mechanistic flexibility.

Despite their apparent mutational tolerance, several of these sites are highly conserved across Group M HIV-1 strains (Supplementary data 3). For example, residue 13 is conserved as valine in 99.6% of sequences, and residue 14 as aspartate in 99.8%. Both fall within the overlapping reading frame shared between *vif* and the C-terminal tail of *pol*-encoded IN (Fig. 1A). However, this overlap was removed in the proviral construct harboring the DMS library. Thus, their evolutionary conservation in native HIV-1 genomes may primarily reflect constraints imposed by the *pol* reading frame, rather than direct selective pressures imposed by A3G on Vif.

Twelve of the 24 ‘mutationally tolerant’ residues map to established interfaces with A3G, RNA, and/or CBFβ (Fig. 3A, dashed lines) (26). Mutational tolerance at such interface residues is unexpected but may reflect evolved structural robustness that accommodates amino acid variation without loss of function. To investigate this possibility, we focused on position 42, a perfectly conserved histidine (H) across HIV-1 and SIV_cpz_ that binds RNA that bridges Vif and A3G (26). Despite its conservation, this site appeared highly mutationally tolerant in the DMS LAI *vif* dataset (Fig. 3A, star; Fig. 3B, red line marks the library-wide median). Notably, several enriched substitutions (N, D, P, R, and L) are accessible via single-nucleotide changes, highlighting the potential for adaptive variation even at canonical interface residues.

To assess whether this tolerance reflects true functional flexibility, we performed an orthogonal A3G packaging assay (Fig. 3C). Individual missense variants at LAI Vif position 42 were cloned into a lentiviral expression vector and co-transfected into HEK293T cells with FLAG-tagged A3G. Seventy-two hours post-transfection, virions were harvested and A3G incorporation was quantified by western blot (Fig. 3C). Functional outcomes from this single-variant assay largely recapitulated the pooled DMS data (Fig. 3B). Substitutions with the lowest enrichment scores, such as H42I and H42G (−3.20 and −3.22, respectively; Fig. 3B), led to markedly elevated A3G packaging relative to WT Vif (Fig. 3C), indicating substantial loss of function. Moderately depleted variants like H42V and H42Y (average log enrichment values of −0.68 and −0.78, respectively, Fig. 3B) also showed partial functional defects (Fig. 3C). In contrast, H42A, H42P, H42K, and H42N (average log enrichment ratios of −0.04, −0.29, 0.29, and 1.02, respectively) excluded A3G nearly as effectively as WT Vif (Fig. 3C). A few variants (*e.g*., H42T and H42D) showed discordant behavior: although enriched in the DMS assay, they failed to exclude A3G in the packaging assay. This discrepancy may reflect partial impairment not detected by pooled selection, context-specific effects, or noise from the DMS experiment. Overall, these results confirm that position 42 tolerates many missense substitutions for A3G antagonism despite its strict evolutionary conservation. This suggests that conservation at this site may reflect historical contingency or overlapping functional constraints, rather than strict biochemical necessity for A3G antagonism.

### Vif constraints and adaptive potential differ between two divergent HIV-1 strains

**Figure 4:**
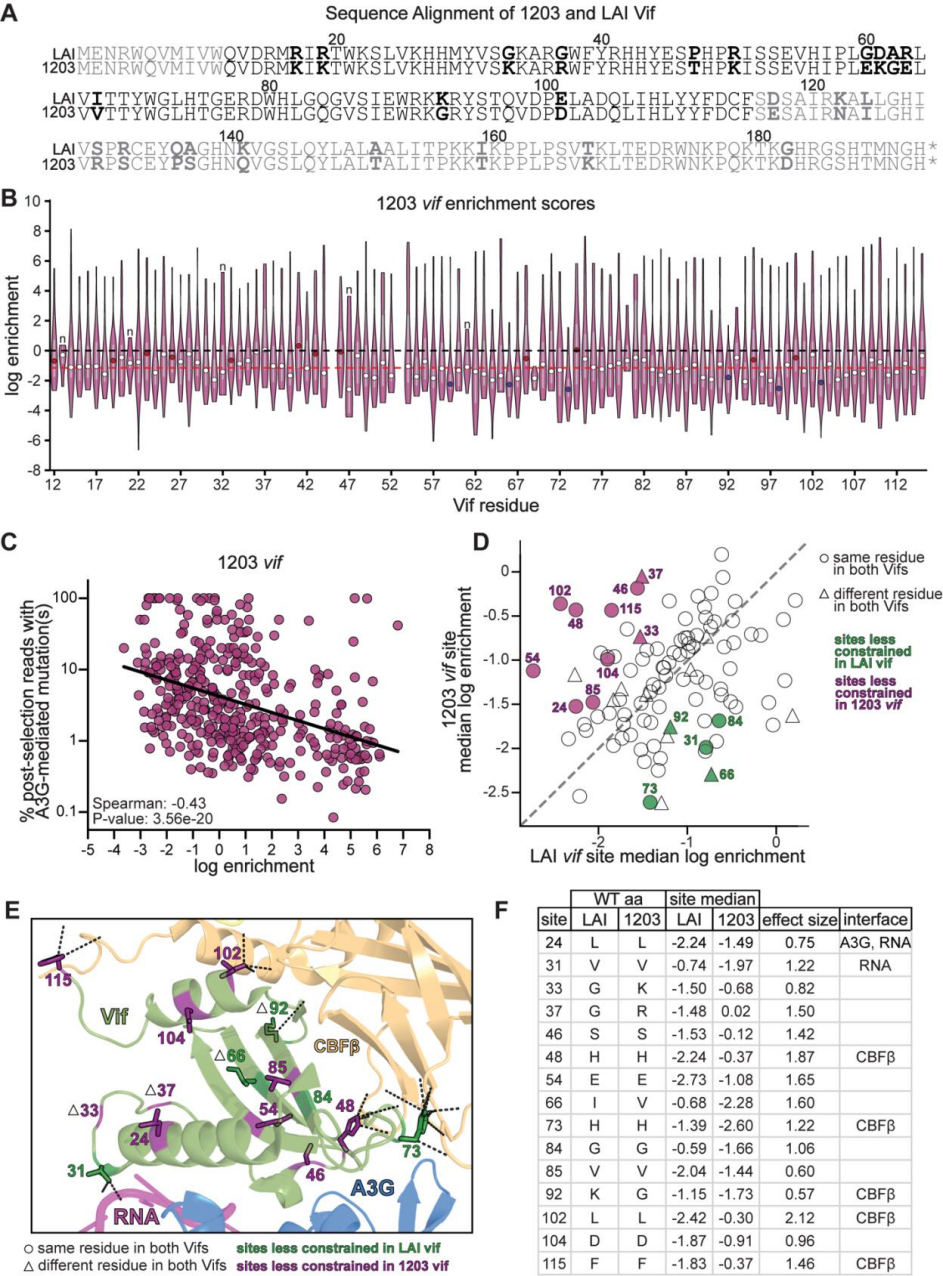
Comparative DMS reveals strain-specific constraints and adaptive divergence between LAI and 1203 Vif proteins A. Amino acid sequence alignment of HIV-1 LAI and 1203 Vif proteins. Divergent residues are bolded in black. Positions not included in the DMS libraries are shown in gray. B. Violin plots showing the distribution of log_2_ enrichment ratios across residues 12–115 in the 1203 Vif DMS library following selection in SupT1-A3G cells. Filled red (higher) and blue (lower) circles indicate sites with significant deviation from the library-wide median (Benjamini–Hochberg FDR q ≤ 0.10 by either a two-sided Wilcoxon test or a direction-matched one-sided test). Sites with <5 missense variants are labeled “n”. C. Scatter plot comparing each variant’s log_2_ enrichment ratio with the frequency of A3G-mediated G-to-A mutations at predicted target motifs. A significant negative correlation (Spearman ρ = −0.43, p = 3.56e–20) supports the use of enrichment scores as a proxy for A3G antagonism. D. Comparison of site-level median log_2_ enrichment scores between LAI and 1203 Vif DMS datasets (Spearman ρ = 0.243, p = 0.0138). Sites showing significant differences in mutational tolerance between strains (two-sided Mann–Whitney U test, uncorrected p < 0.05) are highlighted in green (less constrained in LAI) or purple (less constrained in 1203). Triangles mark positions where the LAI and 1203 sequences differ. E. Structural mapping of the 15 divergent sites onto the Vif–A3G–VCBC–RNA cryo-EM structure (PDB: 8CX0). Green and purple circles indicate sites with reduced constraint in LAI and 1203, respectively. Dashed lines denote known interaction interfaces. F. Table summarizing divergent sites, including wild-type residues in LAI and 1203, median enrichment scores, and associated interaction interfaces.

To investigate how evolutionary pressures shape the mutational landscape of Vif, we compared DMS profiles from LAI to a divergent HIV-1 clade B strain 1203. Although their *vif* genes share 169 of 192 residues (88% identity; Fig. 4A), the strains differ markedly in origin and antiviral activity. LAI is a laboratory-adapted strain that efficiently antagonizes A3G but exhibits limited activity against A3H (80). In contrast, 1203 Vif antagonizes both A3G and A3H (80), with residues 45–63 and 90–93 previously shown to be particularly important for A3H antagonism (52, 81).

We performed three replicates of our DMS selection assay using the 1203 *vif* sequence (Fig. 4B). Similar to the LAI DMS, we found a moderate concordance between different 1203 replicates with Spearman correlation coefficients (ρ) ranging from 0.45 to 0.53 (Supplementary Figure 1B). In contrast, there was much lower concordance between the LAI and 1203 DMS datasets with a Spearman correlation coefficient (ρ) between 0.00 and 0.17 (Supplementary Figure 1C). Using the same normalization as with LAI, we calculated enrichment scores based on median values across all three replicates. We then compared the resulting enrichment scores with A3G-specific G-to-A mutation frequencies (Fig. 4C). As with DMS LAI *vif* (Fig. 2B), we observed a moderate inverse correlation between A3G-induced mutation burden and variant enrichment (Spearman ρ = −0.43), confirming that the enrichment scores accurately reflect A3G antagonism.

We identified 12 sites with median log enrichment ratios above the library-wide average (Fig. 4B, red circles). One of these, residue 19, typically overlaps with the *pol* stop codon. In natural isolates, depending on whether *pol* terminates with TAG, TAA, or TGA, the overlapping *vif* codon could encode AGX (Serine/Arginine), AAX (Asparagine/Lysine), or GAX (Aspartate/Glutamate). However, because TGA is never used as the *pol* stop codon, site 19 in Vif is almost always restricted to lysine (K, 7.5%), asparagine (N, 17.6%), arginine (R, 73.6%), or serine (S, 1.2%) (Supplementary data). In contrast, our DMS analysis revealed high enrichment scores at site 19 for multiple additional substitutions, suggesting that mutational tolerance at this site increases when the overlap with *pol* is removed. The only strong constraints were against acidic residues: both aspartate and glutamate variants were strongly depleted (average log enrichment of −2.02 and −1.28, respectively), consistent with prior evidence that site 19 contributes to the dual A3G-RNA interface (26). These observations suggest that the requirement to exclude aspartate/glutamate at Vif site 19 may also have simultaneously constrained *pol* from evolving a TGA (opal) stop codon. Thus, the overlap between *vif* and *pol* reciprocally constrains both open reading frames.

We next compared the enrichment profiles of the LAI and 1203 *vif* DMS libraries at the site-level. For each residue, log enrichment values were averaged within each strain, and distributions between LAI and 1203 were compared using two-sided Mann–Whitney U tests (p < 0.05, uncorrected). Sites were classified based on the direction of median difference (“higher in LAI” or “higher in 1203”). Globally, the two Vif proteins exhibited nearly identical levels of constraint, with median log enrichment scores of −1.23 for LAI and −1.14 for 1203. Despite this overall similarity, 15 residues displayed significantly different mutational tolerances between strains (Fig. 4D). Four of these positions (33, 37, 66, and 92) correspond to sites where LAI and 1203 encode different WT amino acids (Fig. 4A; 4D–E, triangles; 4F), suggesting these differences might reflect strain-specific optimization of Vif function under distinct selective pressures. Notably, the remaining eleven positions are identical in sequence between LAI and 1203 Vif yet exhibit divergent mutational tolerance. This observation underscores the role of evolutionary contingency and epistasis in shaping the Vif fitness landscape across strains.

Five of the 15 Vif sites with significantly divergent mutational tolerance between LAI and 1203 map to residues that directly contact CBFβ (26) (Fig. 4E). Given the central role of CBFβ in stabilizing Vif and facilitating A3G antagonism (17, 82, 83), this divergence suggests that Vif has fine-tuned its interaction with CBFβ through distinct evolutionary pathways in different HIV-1 strains. We also identified one site (residue 24) that contacts both A3G and RNA, and another (residue 31) located at the RNA-binding interface (26), both of which showed strain-specific differences in mutational tolerance (Fig. 4E). The presence of such constraints at these functional hotspots supports the hypothesis that Vif has evolved along multiple genotype-dependent trajectories to preserve A3G antagonism. These findings illustrate how host-specific selective pressures, such as the presence of active A3H in the host of 1203, might drive alternative molecular solutions to a conserved functional challenge like A3G. Collectively, these results underscore Vif’s evolutionary plasticity and its capacity to accommodate strain-specific adaptations while safeguarding core functions like A3G antagonism.

**Figure 5.**
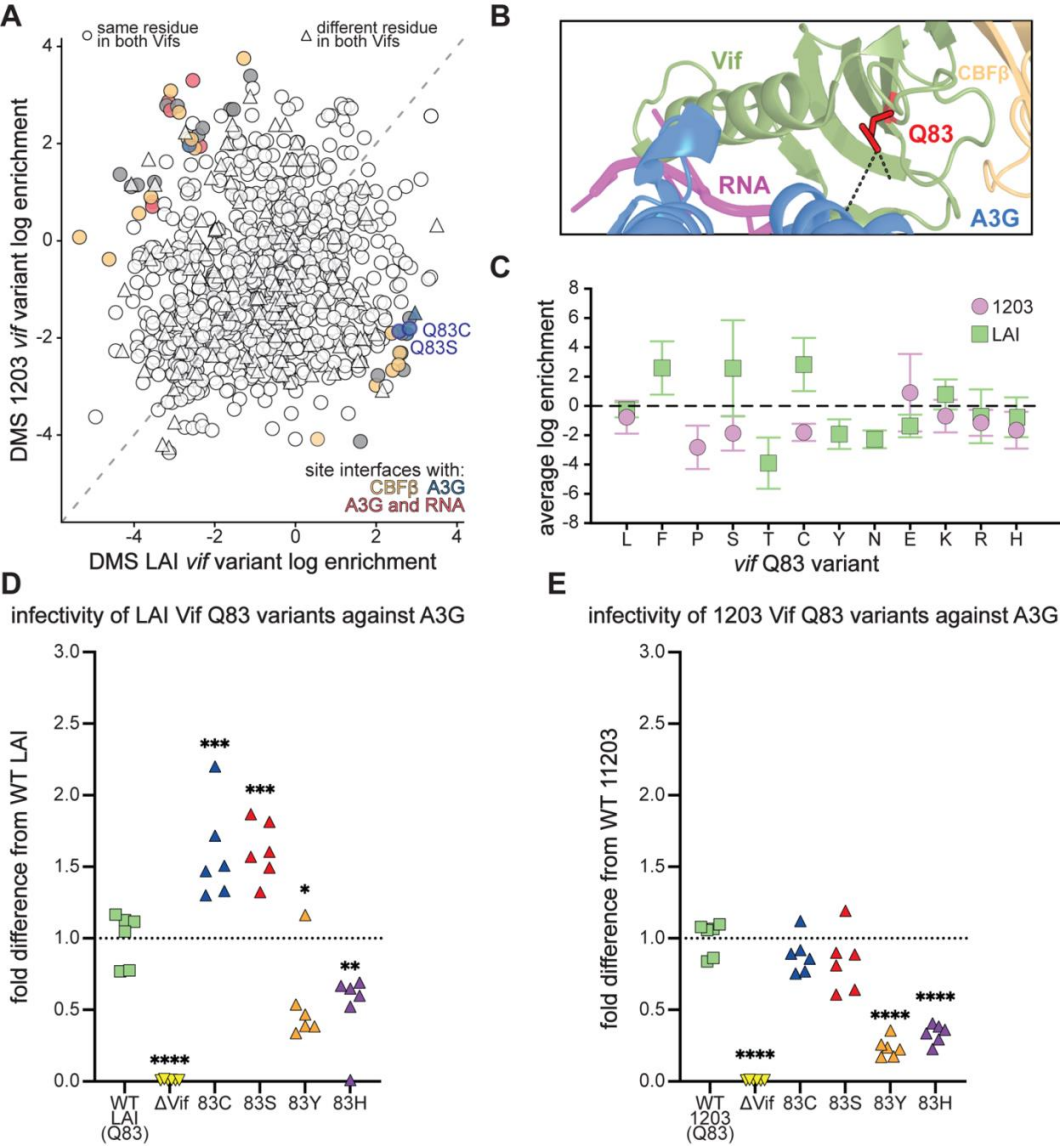
Strain-specific fitness effects of Q83 variants reveal divergent adaptive landscapes in LAI and 1203 Vif. A. Scatter plot comparing average log_2_ enrichment scores of all single amino acid Vif variants between the LAI and 1203 DMS libraries. Variants with strain-specific fitness effects (≥2 standard deviations from the mean of the log_2_[LAI/1203] distribution) are colored according to their mapped interaction interface: A3G (blue), CBFβ (orange), or A3G and RNA (red). Q83C and Q83S are highlighted. B. Structural depiction of Q83 (red) in the LAI Vif–A3G–VCBC–RNA cryo-EM complex (PDB: 8CX0), showing its location at the A3G-binding interface. C. Average log_2_ enrichment scores for all Q83 substitutions in LAI (green squares) and 1203 (purple circles) Vif DMS libraries. The dashed line at 0 indicates the enrichment score of the wild-type residue. Note: enrichment scores are derived from pooled deep sequencing following two rounds of replication in SupT1-A3G cells and are directly comparable to those shown in Fig. 3B. D–E. Infectivity assays for replication-incompetent, VSV-G pseudotyped HIV-1 viruses encoding Q83 variants in the LAI (D) or 1203 (E) Vif background. Infectivity was measured in SupT1 cells expressing physiological levels of A3G and normalized within each experiment to a no-A3G vector control. Bars represent mean fold change in infectivity relative to wild-type Vif in the presence of A3G. Wild-type and ΔVif viruses were included as controls. Error bars represent the standard deviation across biological replicates. Statistical significance was assessed using one-way ANOVA followed by Dunnett’s multiple comparisons test, comparing each variant to wild-type Vif: ns = not significant (P > 0.05); *P ≤ 0.05; **P ≤ 0.01; ***P ≤ 0.001; ****P ≤ 0.0001.

Although site-level analyses provide a broad view of mutational constraint, they can mask important differences in the functional impact of individual missense substitutions. To resolve these finer-scale effects, we next compared the mutational tolerances of all missense variants between the LAI and 1203 *vif* DMS libraries. For each missense variant, we first calculated the mean log enrichment ratio across biological replicates within each strain. We then quantified interstrain differences by subtracting these averages, computed as *log(LAI) − log(1203)*, yielding a Δlog enrichment value for each variant. Thus, positive values indicate variants that are more tolerated in LAI, whereas negative values indicate variants that are more tolerated in 1203. To visualize relative magnitudes, linear fold-changes were obtained as *2^(Δlog enrichment)*. Using this approach, we identified 55 variants across 38 unique Vif residues with significantly divergent functional effects between the two strains, defined as values exceeding two standard deviations from the mean of the Δlog enrichment distribution (Fig. 5A, colored points). Of these 38 residues, five map to the A3G-binding interface (blue points), six contribute to both A3G and RNA interactions (red points), and 17 interact with CBFβ (orange points).

We focused on position 83, a well-characterized residue within Vif’s “arms race interface” with A3G (23, 25, 59). This was the only of four A3G-interacting sites where multiple individual missense variants showed significantly divergent functional effects between the LAI and 1203 *vif* backgrounds. Prior studies demonstrated that the ancient Y83H substitution was pivotal for cross-species transmission of a precursor SIV into hominids, ultimately enabling the emergence of HIV-1 (25). Analysis of *vif* sequences from four representatives of each HIV-1 Group M subtype, including circulating recombinant forms, revealed that position 83 most commonly encodes histidine (H) in 68.5% of sequences, followed by glutamine (Q) in 29.9%, and asparagine (N) in 1.4% (Supplementary data 3). In contrast, clade B viruses display a different preference: Q is strongly favored (93.0%), while H (5.8%) and N (0.8%) occur only rarely (Supplementary data 4, Supplementary Figure 2). LAI and 1203, both clade B strains, encode a Q at this site. Consistent with earlier studies, the Q83Y variant, a reversion to the ancestral state, was strongly depleted in the DMS LAI *vif* dataset (average log enrichment ratio −1.93; Fig. 5C); coverage for this variant was insufficient in the 1203 DMS dataset to make a firm conclusion. Surprisingly, the historically adaptive Q83H variant was also fitness-impaired in both strains, with log enrichment ratios of −0.77 in LAI and −1.63 in 1203 (Fig. 5C).

We performed independent infectivity assays with pseudotyped viruses encoding these Vif variants in either the LAI or 1203 Vif background to confirm these effects. We included a ΔVif virus, which does not express a functional *vif* gene, as a negative control. We found that both Q83Y and Q83H viruses exhibited significantly reduced A3G antagonism compared to wild-type in both strain backgrounds (Fig. 5D–E). The functional impairment of Q83H, along with its low frequency among clade B viruses, indicates a clade B-specific evolutionary constraint that disfavors reversion to histidine. These findings underscore the dynamic nature of host-virus coevolution: residues once essential for cross-species adaptation to overcome human A3G can later become selectively disfavored, due to epistatic interactions with subsequent lineage-specific mutations.

Our analyses of mutations with divergent fitness effects between LAI and 1203 Vif highlighted two notable substitutions at site 83: Q83C and Q83S. Q83C emerged from the DMS LAI *vif* dataset as the most enriched variant at this position (average log enrichment 2.88). Although Q83C has not previously been reported in primary HIV-1 isolates, it was shown to enhance antagonism of a divergent A3G in an *in vivo* experimental evolution study (84). The second most enriched variant from the LAI Vif DMS dataset was Q83S (average log enrichment 2.57). In stark contrast, both Q83C and Q83Y variants were strongly depleted in the DMS 1203 *vif* dataset, with average log enrichment ratios of −1.80 (Q83C) and −1.87 (Q83S) (Fig. 5C). To validate these strain-specific fitness effects, we included these substitutions in our independent infectivity assays with pseudo-typed viruses, allowing us to assess the full impact of each substitution across the entire viral lifecycle. In the LAI Vif background, both Q83C and Q83S conferred significantly enhanced antagonism of A3G compared to wild-type Vif, as expected (Figure 5D). Conversely, in the 1203 Vif background, neither Q83C nor Q83S conferred any advantage over wild-type (Figure 5E). Together, these results reveal a striking example of epistasis, where the fitness consequences of individual substitutions depend strongly on the Vif sequence background. This underscores the strain-specific nature of adaptive potential at the Vif-A3G interface, highlighting how evolutionary trajectories are contingent upon evolutionary history and genetic context.

## Discussion

Our study provides a comprehensive, high-resolution map of the evolutionary constraints and adaptive potential of HIV-1 Vif, the primary antagonist of the host restriction factor APOBEC3G (A3G) and other APOBEC3 proteins. By applying deep mutational scanning (DMS) to *vif* from two divergent HIV-1 strains, we uncovered both mechanistic insights into Vif function and broader principles governing viral antagonism of host innate immunity. Importantly, we found a strong inverse correlation between enrichment scores and A3G-mediated mutation rates, reinforcing DMS as a robust proxy for viral fitness in the presence of A3G and demonstrating its utility for identifying previously uncharacterized, functionally competent Vif variants. More broadly, our approach establishes DMS as a scalable strategy for mapping mutational landscapes across diverse accessory proteins, even in contexts where host-encoded enzymes like A3G introduce additional mutations independent of the experimental design.

To place these findings in an evolutionary context, we compared site-level median enrichment scores from our DMS analysis of A3G antagonism with residue conservation across HIV-1 Group M, including circulating recombinant forms, using the curated Vif subtype alignment from the Los Alamos National Laboratory HIV Sequence Database (Supplementary Data 3). We observed very little correlation between mutational tolerance in our assay and evolutionary conservation, whether in LAI or 1203 (Supplementary Figure 3). This disconnect extended across most residues in both datasets, suggesting that sites tolerant of mutation *in vitro* are often highly conserved *in vivo*. One interpretation is that evolutionary conservation integrates multiple selective pressures — including evasion of several A3 proteins, interactions with cellular cofactors, and overlapping reading frame constraints — whereas our DMS assay isolates the contribution of A3G antagonism in a controlled genetic background. An additional explanation is that natural HIV-1 sequences are not at evolutionary equilibrium. Due to strong phylogenetic relatedness among sequences, even sites that tolerate multiple amino acids may show very limited observed variability. Indeed, a previous DMS study of the HIV envelope protein found only modest correlations between DMS-derived amino acid preferences and natural frequencies; phylogenetic structure alone could constrain observed variability, even when mutational tolerance was accurately measured (46). Thus, even when enrichment scores provide a high-resolution, experimentally tractable view of A3G-specific functional constraints, they still may not fully explain natural sequence conservation *in vivo* due to a combination of historical contingency, structural dependencies, and overlapping selective forces.

Within this fitness landscape, many Vif residues exhibited signs of purifying selection, with substitutions at conserved motifs — such as those required for CBFβ binding or within well-characterized functional domains — most severely compromising activity. Yet we also observed pockets of unexpected tolerance, even at residues embedded within A3G, RNA, and CBFβ binding interfaces (Fig. 3A). These sites highlight Vif’s ability to accommodate alternative sequence states while retaining activity, a hallmark of its evolutionary plasticity. Crucially, by comparing two Vif proteins shaped by distinct evolutionary contexts and selective pressures, we identified multiple positions where the same substitution produced divergent effects in LAI versus 1203 (Fig. 5). This strain-specific epistasis illustrates the highly dynamic nature of the Vif-A3G arms race, in which evolutionary trajectories depend strongly on genetic background. Such contrasts could only be captured through parallel DMS across divergent Vif proteins, underscoring the value of comparative approaches for dissecting host-virus conflict.

Differences in mutational tolerance were observed at several sites in Vif binding interfaces with CBFβ, A3G, and RNA, potentially reflecting distinct host pressures shaping the evolution of LAI and 1203. LAI Vif efficiently antagonizes A3G but not A3H, whereas 1203 Vif can counteract both (80). Vif also mediates degradation of the host phosphatase PP2A (35–37), a function absent in LAI due to a glycine at position 33 (35) but likely present in 1203, which encodes a lysine at this site. Indeed, 1203 Vif closely resembles a primary isolate capable of PP2A antagonism (37–39), differing only by a residue (valine at site 31 rather than isoleucine) that does not abolish this function (37). Thus, while LAI Vif appears optimized for A3G antagonism alone, 1203 Vif must balance activity against A3G, A3H, and likely PP2A. Such expanded functionality may impose additional constraints on Vif, which might restrict its optimization for any single target and explain why mutations tolerated in LAI can be deleterious in 1203, or vice versa. Together, these results support the idea that host genetic variation contributes to strain-specific Vif fitness landscapes and may drive divergent paths of viral adaptation.

Position 83 provides a vivid example of these dynamics. The ancient Y83H substitution enabled cross-species transmission from simian hosts to humans (25), yet in our assays, Q83H was impaired in both LAI and 1203 (Fig. 5C/D/E), consistent with its low frequency among clade B isolates. These findings emphasize that residues once critical for adaptation may later become constrained by lineage-specific pressures. Conversely, Q83C and Q83S enhanced A3G antagonism in LAI but were deleterious or neutral in 1203 (Fig. 5C/D/E), demonstrating strong context dependence.

Although our DMS data revealed many variants with positive enrichment scores, most are absent from circulating HIV-1 strains. While some of these may reflect context-specific gains in fitness, others could arise from experimental noise, sampling variance, or artifacts of pooled competition. Additional validation will be required to distinguish true adaptive variants from false positives. One notable exception is Q83C, which emerged in our dataset as highly enriched and was previously shown to enhance Vif-mediated antagonism of a divergent A3G *in vivo* (84). It remains unclear whether this activity stems from reinforcement of known contacts at the RNA/A3G binding interfaces or the emergence of novel interactions. Importantly, Q83C is one of the few DMS-enriched variants with demonstrated *in vivo* benefit. In contrast, enriched variants at other positions, such as site 42, excluded A3G packaging into virions comparably to wild type *in vitro* (Fig. 3C) but are not sampled in nature (26), suggesting additional constraints not captured in our assays. Together, these observations underscore that while depleted variants reliably signal functional disruption, enriched variants should be interpreted cautiously, particularly in the absence of *in vivo* or orthogonal validation (85).

Finally, our experimental design relieved mutational constraints imposed by the overlapping reading frame shared between *vif* and the C-terminal tail of IN, which is critical for viral integration *in vitro* and *in vivo* (86, 87) and for stabilizing the intasome complex (88). In the DMS LAI *vif* dataset, sites 13 and 14 exhibited higher-than-average median log enrichment scores (Fig. 2A), suggesting that several substitutions were tolerated and may even moderately enhance A3G antagonism. Notably, these residues are almost universally conserved as valine and aspartate, respectively, across HIV-1 Group M (Supplementary Data 3). Our analyses suggest that this conservation reflects constraints from *pol* rather than intrinsic intolerance within *vif*. A similar trend appears at position 19, which overlaps with the IN stop codon and likewise showed higher-than-average median log enrichment in the DMS 1203 *vif* library (Fig. 4B). By decoupling *vif* from the overlapping *pol* reading frame, our experimental design suggests the presence of a broader mutational landscape across positions 13–19 that is inaccessible to HIV evolution in its native genomic context.

Collectively, our findings establish a validated DMS assay for HIV-1 Vif, enabling high-throughput, quantitative analysis of variant fitness in the presence of A3G. By combining pooled functional selections with targeted validation assays, we reveal the delicate balance Vif must strike to maintain effective A3G antagonism while accommodating competing pressures from other host factors and from viral genome architecture. Our approach highlighted both well-established constrained motifs and revealed previously uncharacterized, functionally competent variants, including Q83C. It also revealed mutational tolerance at positions historically conserved due to overlapping reading frame constraints, offering insight into evolutionary plasticity not readily accessible through sequence analysis alone. Beyond HIV, this work illustrates how DMS can disentangle the contributions of individual host pressures from the broader set of evolutionary forces acting on viral accessory proteins, offering a framework for identifying vulnerabilities that arise from the evolutionary compromises inherent to viral fitness.

## Materials & Methods

### Cell culture and transfections

SUPT1 cells stably expressing A3G at physiological levels were a gift from Reuben Harris and Judd Hultquist, as previously described (60). These cells were maintained in RPMI-1640 medium (Gibco, #11875093) supplemented with 10% fetal bovine serum (FBS; GE Healthcare, #SH30910.03), 1% penicillin–streptomycin (Gibco, #15140122), and 1% HEPES at 37°C with 5% CO_2_. Unmodified SUPT1 cells (ATCC CRL-1942) used in validation assays were cultured in RPMI-1640 supplemented with 10% FBS, 1% Antibiotic–Antimycotic (Gibco, #15240062), 1% sodium pyruvate (Gibco, #11360–070), 1% glucose (Gibco, #A24940–01), and 1% GlutaMAX (Gibco, #35050–061). HEK293T cells (ATCC CRL-3216) were maintained in DMEM (Gibco, #11965092) supplemented with 10% FBS and 1% penicillin–streptomycin. HEK293T-c17 cells (ATCC CRL-11268) were maintained in DMEM with 10% FBS and 1% Antibiotic–Antimycotic.

### Plasmids

The plasmid encoding 1203 Vif was described previously (80). Site-directed point mutations for infectivity experiments were introduced using PrimeSTAR HS DNA Polymerase (Takara, #R010B) or synthesized as gene fragments (Twist Bioscience, San Francisco, CA). Mutated fragments were inserted into the HIV-1 LAI-based molecular clone pLAIΔVifΔEnvLuc2 using MluI and XbaI restriction sites, as previously described (25, 80). For packaging assays, position 42 variants were cloned into pHIV packaging vectors and co-transfected with psPAX2 and pMD2.G plasmids (gifts from Didier Trono; Addgene plasmids #12260 and #12259, respectively). The codon-mutant DMS libraries for LAI and 1203 Vif were cloned into a replication-competent HIV-1 proviral backbone in which the endogenous vif start codon overlapping with pol was ablated, as described previously (21). Libraries were digested with MluI and XbaI and ligated into this backbone to generate DMS LAI Vif HIV-1 and DMS 1203 Vif HIV-1 constructs. All plasmid sequences were confirmed by Sanger sequencing (Fred Hutch Genomics and Bioinformatics Shared Resource).

### Construction of DMS *vif* libraries

Deep mutational scanning (DMS) libraries were designed to encode all possible single amino acid substitutions across residues 12–115 of the *vif* genes from HIV-1 LAI and 1203 strains. For each codon, degenerate sequences were selected to encode the desired substitutions while minimizing the introduction of A3G-preferred deamination motifs (e.g., 5′-CC) when alternative codon choices were available. Oligonucleotide pools were synthesized by Twist Bioscience (San Francisco, CA, USA). Variant codons for each position were synthesized as gene fragments flanked by MluI and XbaI restriction sites and delivered in a 96-well plate format, with each well corresponding to a specific residue. For each site, codons encoding the desired amino acid substitutions were pooled in equal proportions. The variant pools for all targeted positions were then combined in equal amounts to generate the complete DMS *vif* libraries for LAI and 1203. These libraries were subsequently cloned into the replication-competent HIV-1 backbone described above.

### Screening DMS *vif* viruses

For each of three biological replicates, 3.1 × 10^7^ SupT1-A3G cells were infected at an MOI of 0.01 by spinoculation (1100 × g, 30 min) in the presence of 20 μg/mL DEAE-dextran. The media was replaced immediately and again 24 hours post-infection. At 72 hours, the supernatant was harvested, supplemented with 20 μg/mL DEAE-dextran, and used to infect 1.5 × 10^7^ fresh SupT1-A3G cells by spinoculation. Following a second 72-hour infection, the post-selection supernatant was collected. For sequencing, supernatants were filtered (0.2 μm) and concentrated using a SW28 rotor (1 h, 4 °C). Viral pellets were resuspended in PBS, and viral RNA was extracted using the Quick-RNA Viral Kit (Zymo Research, #R1035).

### Sequencing analysis of viral supernatants

Viral RNA was reverse transcribed with SuperScript IV (Thermo Fisher #18090010), and *vif* cDNA was amplified by RT-PCR using primers annealing outside the mutagenized region. Amplicons were processed for library preparation as described in (47). Multiplexed libraries were pooled and sequenced on an Illumina NextSeq P2 platform using 250 paired-end reads (Fred Hutch Genomics and Bioinformatics Shared Resource).

### Analysis of DMS results

#### Processing of merged reads and consensus generation

Merged paired-end sequencing reads were processed using custom Python scripts to generate high-confidence consensus sequences for each unique molecular identifier (UMI). Reads were parsed from FASTQ files and filtered to retain only those corresponding to the expected insert length, excluding truncated or over-extended fragments from downstream analysis. For each UMI, nucleotide identities at each position were aggregated across all supporting reads. To generate the consensus call at each base, the nucleotide with the highest associated Phred quality score was selected. This quality-weighted approach prioritizes the most confidently called bases and reduces the influence of low-frequency sequencing errors. The resulting consensus set represented high-confidence full-length coding sequences suitable for codon-level analysis.

#### Generation of codon count tables

High-confidence consensus sequences were translated into codon triplets and mapped to amino acid positions 12–115 of the LAI or 1203 reference Vif sequence. Each codon observed at a given position was tallied to produce codon count tables, where each row corresponded to a codon position in the reference sequence and each column indicated the count of a specific codon variant. The resulting codon count matrices served as the input for subsequent steps of variant identification and enrichment analysis.

#### Site-level enrichment analysis, normalization, and statistical testing

To quantify the selective effects of individual mutations in Vif, codon-level variant counts from pre- and post-selection libraries were converted into relative frequencies. For each variant, the change in frequency after selection was measured by comparing its post-selection abundance to its pre-selection abundance. Pre-selection replicates were pooled to generate a single, high-depth baseline library capturing the full diversity of input variants while minimizing sampling noise. This shared pre-selection reference was used across all post-selection replicates within each strain, ensuring that enrichment calculations reflect biological effects of selection rather than fluctuations in the input distribution. Using a common baseline also improves the statistical power to detect enrichment in low-frequency variants. Because sequencing depth and wild-type representation can vary across codons due to library biases or amplification noise, enrichment ratios were normalized relative to the wild-type codon at each site. This site-specific normalization accounts for local differences in coverage and corrects for site-specific distortions in baseline representation. In effect, each variant’s enrichment score reflects its change relative to the wild-type codon’s behavior under selection. Normalization was only performed at sites where the wild-type codon was sufficiently represented in both pre- and post-selection libraries. For each strain, enrichment ratios were calculated independently for all biological replicates, using the same pooled pre-selection baseline. At each codon position, variant-level enrichments were aggregated by computing the median enrichment across all missense substitutions, yielding a site-level enrichment score. This approach reduces noise from individual variants and captures the overall mutational tolerance or constraint at each residue.

To identify sites under selection within each strain, replicate-level site medians were statistically compared to the global background of enrichment scores using Wilcoxon signed-rank tests. Two complementary tests were performed: two-sided test: detects sites with any deviation (enrichment or depletion) from the global median, one-sided tests: identify sites with consistent enrichment (mutational tolerance) or depletion (functional constraint). False discovery rate (FDR) correction was applied using the Benjamini–Hochberg procedure (FDR ≤ 0.10) to identify statistically significant positions.

#### Identification and quantification of A3G-mutated reads

To identify and quantify A3G-mediated hypermutation in the DMS libraries, consensus FASTQ files were parsed and compared against the reference *vif* sequence in Python. Each read was aligned codon-by-codon to the reference open reading frame, and all single-nucleotide mismatches were recorded along with their position, codon context, and base substitution identity. Substitutions consistent with A3G activity were annotated.

### Functional validation of DMS *vif* variants

Single-cycle infectivity assays were performed as described (80). HEK293T-c17 cells were seeded at 1.5 × 10^5^ cells/ml in 6-well plates and transfected with 400 ng pLAIΔVifΔEnvLuc2 proviral vector, 100 ng L-VSV-G, 3XFlag-tagged A3G or pcDNA4/TO empty vector, and Transit-LT1 (Mirus #MIR2300) at 3.5 μL reagent/μg DNA. Media was replaced after 24 hours, and virus was harvested 72 hours post-transfection, then filtered and normalized by RT-qPCR-based titering (89, 90). Virus inputs were normalized by mock supernatant addition to equalize volumes. Unmodified SupT1 cells were seeded at 1.5 × 10^5^ cells/well in 96-well plates in medium with 20 μg/ml DEAE-dextran. At 72 hours post-infection, cells were lysed in Bright-Glo reagent (Promega #E2610), and luciferase activity was measured on a LUMIstar Omega luminometer (BMG Labtech). Each condition was measured in duplicate technical replicates. Fold difference in infectivity was calculated by dividing the Raw RLUs by the average RLUs for the relevant WT virus (WT LAI or WT 1203). These values were then further normalized by dividing by the mean fold difference in infectivity for *vif* variants tested without A3G (Separate experiment, Supplementary Figure 4).

### Packaging assay validation of H42 variants

HEK293T-c17 cells were seeded at 1.5 × 10^5^ cells/mL in 6-well plates and transfected with 500 ng psPAX2, 100 ng 3×Flag-tagged A3G in pcDNA4/TO, 500 ng pHIV lentivector expressing Vif variants or no Vif, and TransIT-LT1 (Mirus #MIR2300) at 3.5 uL reagent/μg DNA. Media was replaced after 24 hours, and virus was harvested 72 hours post-transfection, then filtered and concentrated by centrifugation (1 h, 4 °C, maximum speed). Viral pellets were resuspended in PBS, normalized by HIV-1 p24 ELISA (R&D #DY7360–05), and prepared for SDS-PAGE using NuPAGE loading dye (Invitrogen #NP007). Proteins were resolved by SDS-PAGE, transferred, and immunoblotted with anti-Flag (Sigma #F1804) or anti-p24 (HIV-ARP #3537), followed by anti-mouse IgG secondary (R&D #HAF007), all at 1:5000. Blots were imaged on a ChemiDoc MP (Bio-Rad) and quantified with ImageJ. A3G incorporation was calculated as the anti-Flag/anti-p24 ratio, normalized to the ΔVif control.

### Analysis of primary isolate Vif sequences

Primary isolate sequences were obtained from curated alignments provided by the Los Alamos National Laboratory HIV sequence database. Group M subtype reference alignments including circulating recombinant forms (CRFs) were used for global analyses. For Clade B–specific analyses, sequences were downloaded using the “one sequence per patient” option in the LANL database interface. Percent frequencies were calculated from alignments of these sequences (Supplementary data)

## Supplementary Material

Supplement 1

Supplement 2

Supplement 3

Supplement 4

Supplement 5

1

## References

[1] Subbramanian R. A., Cohen E. A., Molecular biology of the human immunodeficiency virus accessory proteins. J Virol 68, 6831–6835 (1994). 7933064 10.1128/jvi.68.11.6831-6835.1994 PMC237118

[2] Seelamgari A. , Role of viral regulatory and accessory proteins in HIV-1 replication. Front Biosci 9, 2388–2413 (2004). 15353294 10.2741/1403

[3] Faust T. B., Binning J. M., Gross J. D., Frankel A. D., Making Sense of Multifunctional Proteins: Human Immunodeficiency Virus Type 1 Accessory and Regulatory Proteins and Connections to Transcription. Annu Rev Virol 4, 241–260 (2017). 28961413 10.1146/annurev-virology-101416-041654 PMC5750048

[4] Strebel K., HIV accessory proteins versus host restriction factors. Curr Opin Virol 3, 692–699 (2013). 24246762 10.1016/j.coviro.2013.08.004 PMC3855913

[5] Bergantz L., Subra F., Deprez E., Delelis O., Richetta C., Interplay between Intrinsic and Innate Immunity during HIV Infection. Cells 8 (2019). 10.3390/cells8080922 PMC6721663 31426525

[6] Kirchhoff F., Immune evasion and counteraction of restriction factors by HIV-1 and other primate lentiviruses. Cell Host Microbe 8, 55–67 (2010). 20638642 10.1016/j.chom.2010.06.004

[7] Duggal N. K., Emerman M., Evolutionary conflicts between viruses and restriction factors shape immunity. Nat Rev Immunol 12, 687–695 (2012). 22976433 10.1038/nri3295 PMC3690816

[8] Boso G., Kozak C. A., Retroviral Restriction Factors and Their Viral Targets: Restriction Strategies and Evolutionary Adaptations. Microorganisms 8 (2020). 10.3390/microorganisms8121965 PMC7764263 33322320

[9] Chaipan C., Smith J. L., Hu W.-S., Pathak V. K., APOBEC3G Restricts HIV-1 to a Greater Extent than APOBEC3F and APOBEC3DE in Human Primary CD4 + T Cells and Macrophages. Journal of Virology 87, 444–453 (2013). 23097438 10.1128/JVI.00676-12 PMC3536366

[10] Zhang H. , The cytidine deaminase CEM15 induces hypermutation in newly synthesized HIV-1 DNA. Nature 424, 94–98 (2003). 12808465 10.1038/nature01707 PMC1350966

[11] Harris R. S. , DNA deamination mediates innate immunity to retroviral infection. Cell 113, 803–809 (2003). 12809610 10.1016/s0092-8674(03)00423-9

[12] Mangeat B. , Broad antiretroviral defence by human APOBEC3G through lethal editing of nascent reverse transcripts. Nature 424, 99–103 (2003). 12808466 10.1038/nature01709

[13] Yu Q. , Single-strand specificity of APOBEC3G accounts for minus-strand deamination of the HIV genome. Nat Struct Mol Biol 11, 435–442 (2004). 15098018 10.1038/nsmb758

[14] Sheehy A. M., Gaddis N. C., Malim M. H., The antiretroviral enzyme APOBEC3G is degraded by the proteasome in response to HIV-1 Vif. Nat Med 9, 1404–1407 (2003). 14528300 10.1038/nm945

[15] Conticello S. G., Harris R. S., Neuberger M. S., The Vif protein of HIV triggers degradation of the human antiretroviral DNA deaminase APOBEC3G. Curr Biol 13, 2009–2013 (2003). 14614829 10.1016/j.cub.2003.10.034

[16] Yu X. , Induction of APOBEC3G Ubiquitination and Degradation by an HIV-1 Vif-Cul5-SCF Complex. Science 302, 1056–1060 (2003). 14564014 10.1126/science.1089591

[17] Guo Y. , Structural basis for hijacking CBF-beta and CUL5 E3 ligase complex by HIV-1 Vif. Nature 505, 229–233 (2014). 24402281 10.1038/nature12884

[18] Stanley B. J. , Structural insight into the human immunodeficiency virus Vif SOCS box and its role in human E3 ubiquitin ligase assembly. J Virol 82, 8656–8663 (2008). 18562529 10.1128/JVI.00767-08 PMC2519636

[19] Marin M., Rose K. M., Kozak S. L., Kabat D., HIV-1 Vif protein binds the editing enzyme APOBEC3G and induces its degradation. Nat Med 9, 1398–1403 (2003). 14528301 10.1038/nm946

[20] Mehle A., Goncalves J., Santa-Marta M., McPike M., Gabuzda D., Phosphorylation of a novel SOCS-box regulates assembly of the HIV-1 Vif-Cul5 complex that promotes APOBEC3G degradation. Genes Dev 18, 2861–2866 (2004). 15574592 10.1101/gad.1249904 PMC534646

[21] Etienne L. , The Role of the Antiviral APOBEC3 Gene Family in Protecting Chimpanzees against Lentiviruses from Monkeys. PLoS Pathog 11, e1005149 (2015). 26394054 10.1371/journal.ppat.1005149 PMC4578921

[22] Etienne L., Hahn B. H., Sharp P. M., Matsen F. A., Emerman M., Gene loss and adaptation to hominids underlie the ancient origin of HIV-1. Cell Host Microbe 14, 85–92 (2013). 23870316 10.1016/j.chom.2013.06.002 PMC3733229

[23] Chesarino N. M., Emerman M., HIV-1 Vif Gained Breadth in APOBEC3G Specificity after Cross-Species Transmission of Its Precursors. J Virol 96, e0207121 (2022). 34908448 10.1128/jvi.02071-21 PMC8865550

[24] Compton A. A., Hirsch V. M., Emerman M., The host restriction factor APOBEC3G and retroviral Vif protein coevolve due to ongoing genetic conflict. Cell Host Microbe 11, 91–98 (2012). 22264516 10.1016/j.chom.2011.11.010 PMC3266539

[25] Binning J. M., Chesarino N. M., Emerman M., Gross J. D., Structural Basis for a Species-Specific Determinant of an SIV Vif Protein toward Hominid APOBEC3G Antagonism. Cell Host Microbe 26, 739–747 e734 (2019). 31830442 10.1016/j.chom.2019.10.014 PMC6913891

[26] Li Y. L. , The structural basis for HIV-1 Vif antagonism of human APOBEC3G. Nature 615, 728–733 (2023). 36754086 10.1038/s41586-023-05779-1 PMC10033410

[27] Ito F. , Structural basis for HIV-1 antagonism of host APOBEC3G via Cullin E3 ligase. Sci Adv 9, eade3168 (2023). 10.1126/sciadv.ade3168 PMC9812381 36598981

[28] Kouno T. , Structural insights into RNA bridging between HIV-1 Vif and antiviral factor APOBEC3G. Nat Commun 14, 4037 (2023). 37419875 10.1038/s41467-023-39796-5 PMC10328928

[29] Lewitus E., Li Y., Rolland M., HIV-1 Vif global diversity and possible APOBEC-mediated response since 1980. Virus Evol 11, veae108 (2025). 10.1093/ve/veae108 PMC11781276 39886100

[30] Simon V. , Natural variation in Vif: differential impact on APOBEC3G/3F and a potential role in HIV-1 diversification. PLoS Pathog 1, e6 (2005). 16201018 10.1371/journal.ppat.0010006 PMC1238741

[31] De Maio F. A. , Effect of HIV-1 Vif variability on progression to pediatric AIDS and its association with APOBEC3G and CUL5 polymorphisms. Infect Genet Evol 11, 1256–1262 (2011). 21571098 10.1016/j.meegid.2011.04.020

[32] Ooms M. , HIV-1 Vif adaptation to human APOBEC3H haplotypes. Cell Host Microbe 14, 411–421 (2013). 24139399 10.1016/j.chom.2013.09.006

[33] Peng J. , A naturally occurring Vif mutant (I107T) attenuates anti-APOBEC3G activity and HIV-1 replication. J Mol Biol 425, 2840–2852 (2013). 23707381 10.1016/j.jmb.2013.05.015

[34] Refsland E. W. , Natural polymorphisms in human APOBEC3H and HIV-1 Vif combine in primary T lymphocytes to affect viral G-to-A mutation levels and infectivity. PLoS Genet 10, e1004761 (2014). 25411794 10.1371/journal.pgen.1004761 PMC4238949

[35] Salamango D. J. , HIV-1 Vif Triggers Cell Cycle Arrest by Degrading Cellular PPP2R5 Phospho-regulators. Cell Rep 29, 1057–1065 e1054 (2019). 31665623 10.1016/j.celrep.2019.09.057 PMC6903395

[36] Greenwood E. J. , Temporal proteomic analysis of HIV infection reveals remodelling of the host phosphoproteome by lentiviral Vif variants. Elife 5 (2016). 10.7554/eLife.18296 PMC5085607 27690223

[37] Marelli S. Antagonism of PP2A is an independent and conserved function of HIV-1 Vif and causes cell cycle arrest. Elife 9 (2020). 10.7554/eLife.53036 PMC7920553 32292164

[38] Nagata K., Shindo K., Matsui Y., Shirakawa K., Takaori-Kondo A., Critical role of PP2A-B56 family protein degradation in HIV-1 Vif mediated G2 cell cycle arrest. Biochem Biophys Res Commun 527, 257–263 (2020). 32446377 10.1016/j.bbrc.2020.04.123

[39] Salamango D. J. , Functional and Structural Insights into a Vif/PPP2R5 Complex Elucidated Using Patient HIV-1 Isolates and Computational Modeling. J Virol 94 (2020). 10.1128/JVI.00631-20 PMC7565612 32847850

[40] Iwabu Y. , Differential anti-APOBEC3G activity of HIV-1 Vif proteins derived from different subtypes. J Biol Chem 285, 35350–35358 (2010). 20833716 10.1074/jbc.M110.173286 PMC2975159

[41] Binka M., Ooms M., Steward M., Simon V., The activity spectrum of Vif from multiple HIV-1 subtypes against APOBEC3G, APOBEC3F, and APOBEC3H. J Virol 86, 49–59 (2012). 22013041 10.1128/JVI.06082-11 PMC3255910

[42] Lisovsky I. , HIV-1 subtype variability in Vif derived from molecular clones affects APOBEC3G-mediated host restriction. Intervirology 56, 258–264 (2013). 23689841 10.1159/000348513

[43] Araya C. L., Fowler D. M., Deep mutational scanning: assessing protein function on a massive scale. Trends Biotechnol 29, 435–442 (2011). 21561674 10.1016/j.tibtech.2011.04.003 PMC3159719

[44] Fowler D. M., Fields S., Deep mutational scanning: a new style of protein science. Nat Methods 11, 801–807 (2014). 25075907 10.1038/nmeth.3027 PMC4410700

[45] Fowler D. M., Stephany J. J., Fields S., Measuring the activity of protein variants on a large scale using deep mutational scanning. Nat Protoc 9, 2267–2284 (2014). 25167058 10.1038/nprot.2014.153 PMC4412028

[46] Haddox H. K., Dingens A. S., Bloom J. D., Experimental Estimation of the Effects of All Amino-Acid Mutations to HIV’s Envelope Protein on Viral Replication in Cell Culture. PLoS Pathog 12, e1006114 (2016). 27959955 10.1371/journal.ppat.1006114 PMC5189966

[47] Doud M. B., Bloom J. D., Accurate Measurement of the Effects of All Amino-Acid Mutations on Influenza Hemagglutinin. Viruses 8 (2016). 10.3390/v8060155 PMC4926175 27271655

[48] Dingens A. S., Haddox H. K., Overbaugh J., Bloom J. D., Comprehensive Mapping of HIV-1 Escape from a Broadly Neutralizing Antibody. Cell Host Microbe 21, 777–787 e774 (2017). 28579254 10.1016/j.chom.2017.05.003 PMC5512576

[49] Zhang Z. , Accurate inference of the full base-pairing structure of RNA by deep mutational scanning and covariation-induced deviation of activity. Nucleic Acids Research 48, 1451–1465 (2019). 10.1093/nar/gkz1192 PMC7026644 31872260

[50] Haddox H. K. , Jointly modeling deep mutational scans identifies shifted mutational effects among SARS-CoV-2 spike homologs. bioRxiv 10.1101/2023.07.31.551037 (2023).

[51] Li M. M., Emerman M., Polymorphism in human APOBEC3H affects a phenotype dominant for subcellular localization and antiviral activity. J Virol 85, 8197–8207 (2011). 21653666 10.1128/JVI.00624-11 PMC3147987

[52] Wang J. , The Role of RNA in HIV-1 Vif-Mediated Degradation of APOBEC3H. J Mol Biol 431, 5019–5031 (2019). 31628948 10.1016/j.jmb.2019.09.014 PMC6948013

[53] Desimmie B. A. , Multiple APOBEC3 restriction factors for HIV-1 and one Vif to rule them all. J Mol Biol 426, 1220–1245 (2014). 24189052 10.1016/j.jmb.2013.10.033 PMC3943811

[54] Mehle A. , Identification of an APOBEC3G binding site in human immunodeficiency virus type 1 Vif and inhibitors of Vif-APOBEC3G binding. J Virol 81, 13235–13241 (2007). 17898068 10.1128/JVI.00204-07 PMC2169136

[55] Russell R. A., Pathak V. K., Identification of two distinct human immunodeficiency virus type 1 Vif determinants critical for interactions with human APOBEC3G and APOBEC3F. J Virol 81, 8201–8210 (2007). 17522216 10.1128/JVI.00395-07 PMC1951317

[56] Schröfelbauer B., Senger T., Manning G., Landau N. R., Mutational alteration of human immunodeficiency virus type 1 Vif allows for functional interaction with nonhuman primate APOBEC3G. J Virol 80, 5984–5991 (2006). 16731937 10.1128/JVI.00388-06 PMC1472613

[57] Tian C. , Differential requirement for conserved tryptophans in human immunodeficiency virus type 1 Vif for the selective suppression of APOBEC3G and APOBEC3F. J Virol 80, 3112–3115 (2006). 16501124 10.1128/JVI.80.6.3112-3115.2006 PMC1395459

[58] Compton A. A., Emerman M., Convergence and divergence in the evolution of the APOBEC3G-Vif interaction reveal ancient origins of simian immunodeficiency viruses. PLoS Pathog 9, e1003135 (2013). 23359341 10.1371/journal.ppat.1003135 PMC3554591

[59] Letko M., Booiman T., Kootstra N., Simon V., Ooms M., Identification of the HIV-1 Vif and Human APOBEC3G Protein Interface. Cell Rep 13, 1789–1799 (2015). 26628364 10.1016/j.celrep.2015.10.068 PMC4670588

[60] Hultquist J. F. , Human and rhesus APOBEC3D, APOBEC3F, APOBEC3G, and APOBEC3H demonstrate a conserved capacity to restrict Vif-deficient HIV-1. J Virol 85, 11220–11234 (2011). 21835787 10.1128/JVI.05238-11 PMC3194973

[61] Ooms M., Letko M., Binka M., Simon V., The Resistance of Human APOBEC3H to HIV-1 NL4–3 Molecular Clone Is Determined by a Single Amino Acid in Vif. PLOS ONE 8, e57744 (2013). 23469063 10.1371/journal.pone.0057744 PMC3585137

[62] Haddox H. K., Dingens A. S., Hilton S. K., Overbaugh J., Bloom J. D., Mapping mutational effects along the evolutionary landscape of HIV envelope. eLife 7, e34420 (2018). 29590010 10.7554/eLife.34420 PMC5910023

[63] Rathore A. , The local dinucleotide preference of APOBEC3G can be altered from 5’-CC to 5’-TC by a single amino acid substitution. J Mol Biol 425, 4442–4454 (2013). 23938202 10.1016/j.jmb.2013.07.040 PMC3812309

[64] Vartanian J. P., Meyerhans A., Asjo B., Wain-Hobson S., Selection, recombination, and G----A hypermutation of human immunodeficiency virus type 1 genomes. J Virol 65, 1779–1788 (1991). 2002543 10.1128/jvi.65.4.1779-1788.1991 PMC239985

[65] Janini M., Rogers M., Birx D. R., McCutchan F. E., Human immunodeficiency virus type 1 DNA sequences genetically damaged by hypermutation are often abundant in patient peripheral blood mononuclear cells and may be generated during near-simultaneous infection and activation of CD4(+) T cells. J Virol 75, 7973–7986 (2001). 11483742 10.1128/JVI.75.17.7973-7986.2001 PMC115041

[66] Ho Y. C. , Replication-competent noninduced proviruses in the latent reservoir increase barrier to HIV-1 cure. Cell 155, 540–551 (2013). 24243014 10.1016/j.cell.2013.09.020 PMC3896327

[67] Bruner K. M. , Defective proviruses rapidly accumulate during acute HIV-1 infection. Nat Med 22, 1043–1049 (2016). 27500724 10.1038/nm.4156 PMC5014606

[68] Delviks-Frankenberry K. A. , Minimal Contribution of APOBEC3-Induced G-to-A Hypermutation to HIV-1 Recombination and Genetic Variation. PLoS Pathog 12, e1005646 (2016). 27186986 10.1371/journal.ppat.1005646 PMC4871359

[69] Wilcoxon F., Individual comparisons of grouped data by ranking methods. J Econ Entomol 39, 269 (1946). 20983181 10.1093/jee/39.2.269

[70] Benjamini Y., Yekutieli D., The Control of the False Discovery Rate in Multiple Testing under Dependency. The Annals of Statistics 29, 1165–1188 (2001).

[71] Toledano I., Supek F., Lehner B., Genome-scale quantification and prediction of pathogenic stop codon readthrough by small molecules. Nature Genetics 56, 1914–1924 (2024). 39174735 10.1038/s41588-024-01878-5 PMC11387191

[72] Rubin A. F. , A statistical framework for analyzing deep mutational scanning data. Genome Biol 18, 150 (2017). 28784151 10.1186/s13059-017-1272-5 PMC5547491

[73] Chen G., He Z., Wang T., Xu R., Yu X. F., A patch of positively charged amino acids surrounding the human immunodeficiency virus type 1 Vif SLVx4Yx9Y motif influences its interaction with APOBEC3G. J Virol 83, 8674–8682 (2009). 19535450 10.1128/JVI.00653-09 PMC2738209

[74] Dang Y., Wang X., Zhou T., York I. A., Zheng Y. H., Identification of a novel WxSLVK motif in the N terminus of human immunodeficiency virus and simian immunodeficiency virus Vif that is critical for APOBEC3G and APOBEC3F neutralization. J Virol 83, 8544–8552 (2009). 19535447 10.1128/JVI.00651-09 PMC2738151

[75] Dang Y., Davis R. W., York I. A., Zheng Y. H., Identification of 81LGxGxxIxW89 and 171EDRW174 domains from human immunodeficiency virus type 1 Vif that regulate APOBEC3G and APOBEC3F neutralizing activity. J Virol 84, 5741–5750 (2010). 20335268 10.1128/JVI.00079-10 PMC2876610

[76] Dang Y., Wang X., York I. A., Zheng Y. H., Identification of a critical T(Q/D/E)x5ADx2(I/L) motif from primate lentivirus Vif proteins that regulate APOBEC3G and APOBEC3F neutralizing activity. J Virol 84, 8561–8570 (2010). 20592083 10.1128/JVI.00960-10 PMC2919012

[77] He Z., Zhang W., Chen G., Xu R., Yu X. F., Characterization of conserved motifs in HIV-1 Vif required for APOBEC3G and APOBEC3F interaction. J Mol Biol 381, 1000–1011 (2008). 18619467 10.1016/j.jmb.2008.06.061

[78] Pery E., Rajendran K. S., Brazier A. J., Gabuzda D., Regulation of APOBEC3 proteins by a novel YXXL motif in human immunodeficiency virus type 1 Vif and simian immunodeficiency virus SIVagm Vif. J Virol 83, 2374–2381 (2009). 19109396 10.1128/JVI.01898-08 PMC2643725

[79] Huthoff H., Malim M. H., Identification of amino acid residues in APOBEC3G required for regulation by human immunodeficiency virus type 1 Vif and Virion encapsidation. J Virol 81, 3807–3815 (2007). 17267497 10.1128/JVI.02795-06 PMC1866099

[80] Li M. M., Wu L. I., Emerman M., The range of human APOBEC3H sensitivity to lentiviral Vif proteins. J Virol 84, 88–95 (2010). 19828612 10.1128/JVI.01344-09 PMC2798431

[81] Ooms M., Letko M., Simon V., The Structural Interface between HIV-1 Vif and Human APOBEC3H. J Virol 91 (2017). 10.1128/JVI.02289-16 PMC5309942 28031368

[82] Zhang W., Du J., Evans S. L., Yu Y., Yu X. F., T-cell differentiation factor CBF-beta regulates HIV-1 Vif-mediated evasion of host restriction. Nature 481, 376–379 (2011). 22190036 10.1038/nature10718

[83] Jager S. , Vif hijacks CBF-beta to degrade APOBEC3G and promote HIV-1 infection. Nature 481, 371–375 (2011). 22190037 10.1038/nature10693 PMC3310910

[84] Compton A. A., Malik H. S., Emerman M., Host gene evolution traces the evolutionary history of ancient primate lentiviruses. Philos Trans R Soc Lond B Biol Sci 368, 20120496 (2013). 23938749 10.1098/rstb.2012.0496 PMC3758184

[85] Reeb J., Wirth T., Rost B., Variant effect predictions capture some aspects of deep mutational scanning experiments. BMC Bioinformatics 21, 107 (2020). 32183714 10.1186/s12859-020-3439-4 PMC7077003

[86] Dar M. J. , Biochemical and virological analysis of the 18-residue C-terminal tail of HIV-1 integrase. Retrovirology 6, 94 (2009). 19840380 10.1186/1742-4690-6-94 PMC2770994

[87] Mohammed K. D., Topper M. B., Muesing M. A., Sequential deletion of the integrase (Gag-Pol) carboxyl terminus reveals distinct phenotypic classes of defective HIV-1. J Virol 85, 4654–4666 (2011). 21367893 10.1128/JVI.02374-10 PMC3126176

[88] Li M. , HIV-1 Intasomes Assembled with Excess Integrase C-Terminal Domain Protein Facilitate Structural Studies by Cryo-EM and Reveal the Role of the Integrase C-Terminal Tail in HIV-1 Integration. Viruses 16 (2024). 10.3390/v16071166 PMC11281638 39066328

[89] Vermeire J. , Quantification of reverse transcriptase activity by real-time PCR as a fast and accurate method for titration of HIV, lenti- and retroviral vectors. PLoS One 7, e50859 (2012). 23227216 10.1371/journal.pone.0050859 PMC3515444

[90] Garcia E. I., Emerman M., Recurrent Loss of APOBEC3H Activity during Primate Evolution. J Virol 92 (2018). 10.1128/JVI.00971-18 PMC6096791 29925657

