# Supplementary material for: Fitness Landscapes of APOBEC3G Antagonism by HIV-1 Vif proteins
Source: bioRxiv. 2025 Oct 20:2025.10.20.683452. Preprint. [Version 1] doi: 10.1101/2025.10.20.683452 (PMC12633382; doi:10.1101/2025.10.20.683452)
Supplement: 1 [file NIHPP2025.10.20.683452V1-supplement-1.pdf]

bioRxiv preprint doi: https://doi.org/10.1101/2025.10.20.683452; this version posted October 20, 2025. The copyright holder for this preprint (which was not certified by peer review) is the author/funder, who has granted bioRxiv a license to display the preprint in perpetuity. It is made available under aCC-BY 4.0 International license.

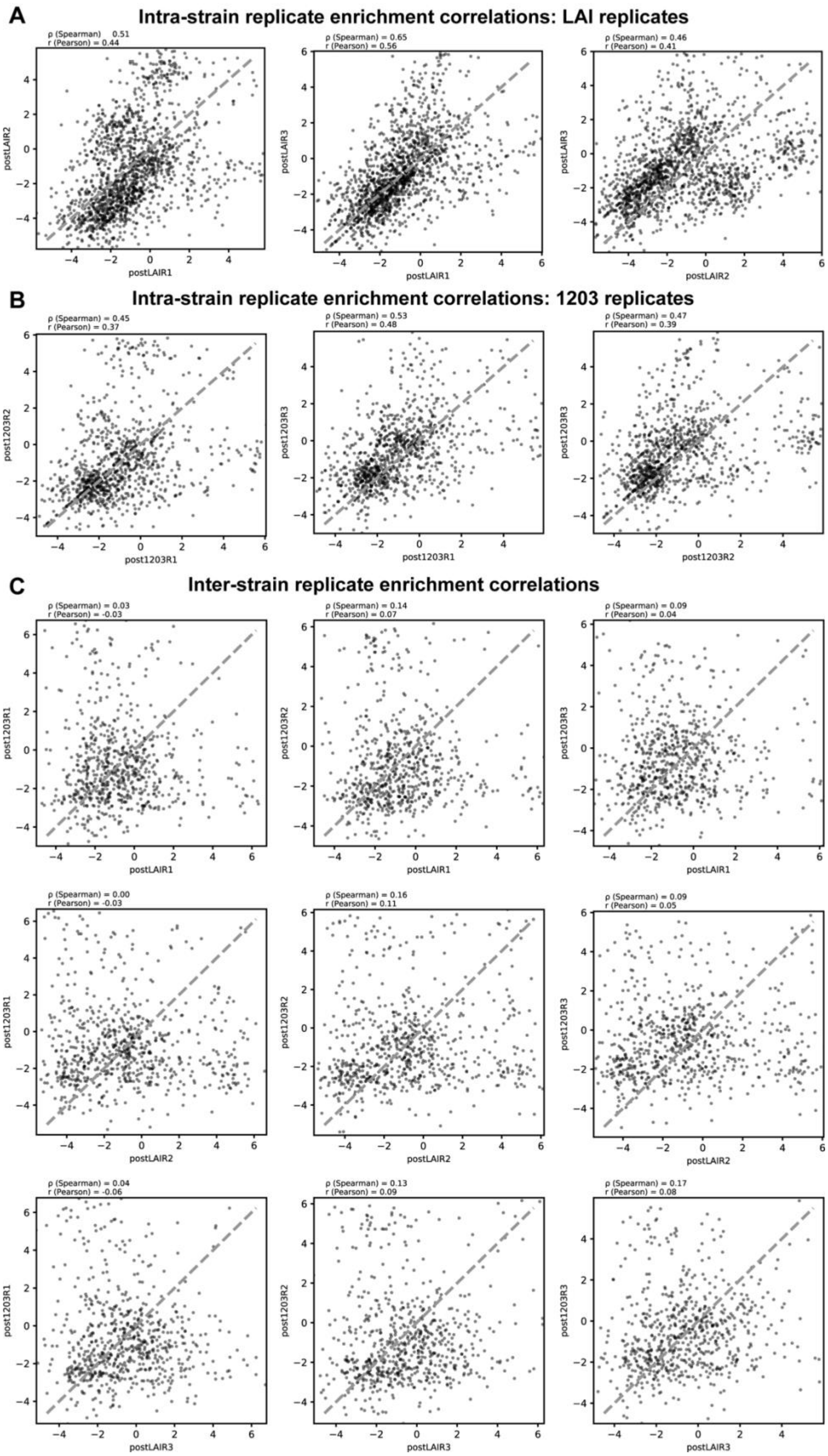

bioRxiv preprint doi: https://doi.org/10.1101/2025.10.20.683452; this version posted October 20, 2025. The copyright holder for this preprint (which was not certified by peer review) is the author/funder, who has granted bioRxiv a license to display the preprint in perpetuity. It is made available under aCC-BY 4.0 International license.

Supplementary Figure 1: Pairwise Spearman correlations of DMS enrichment scores across replicates and strains.
Correlation plots comparing enrichment scores between biological replicates within the same HIV-1 Vif strain (intra-strain) and across different strains (inter-strain). Each point represents a single Vif variant's enrichment score the DMS assay.

bioRxiv preprint doi: https://doi.org/10.1101/2025.10.20.683452; this version posted October 20, 2025. The copyright holder for this preprint (which was not certified by peer review) is the author/funder, who has granted bioRxiv a license to display the preprint in perpetuity. It is made available under aCC-BY 4.0 International license.

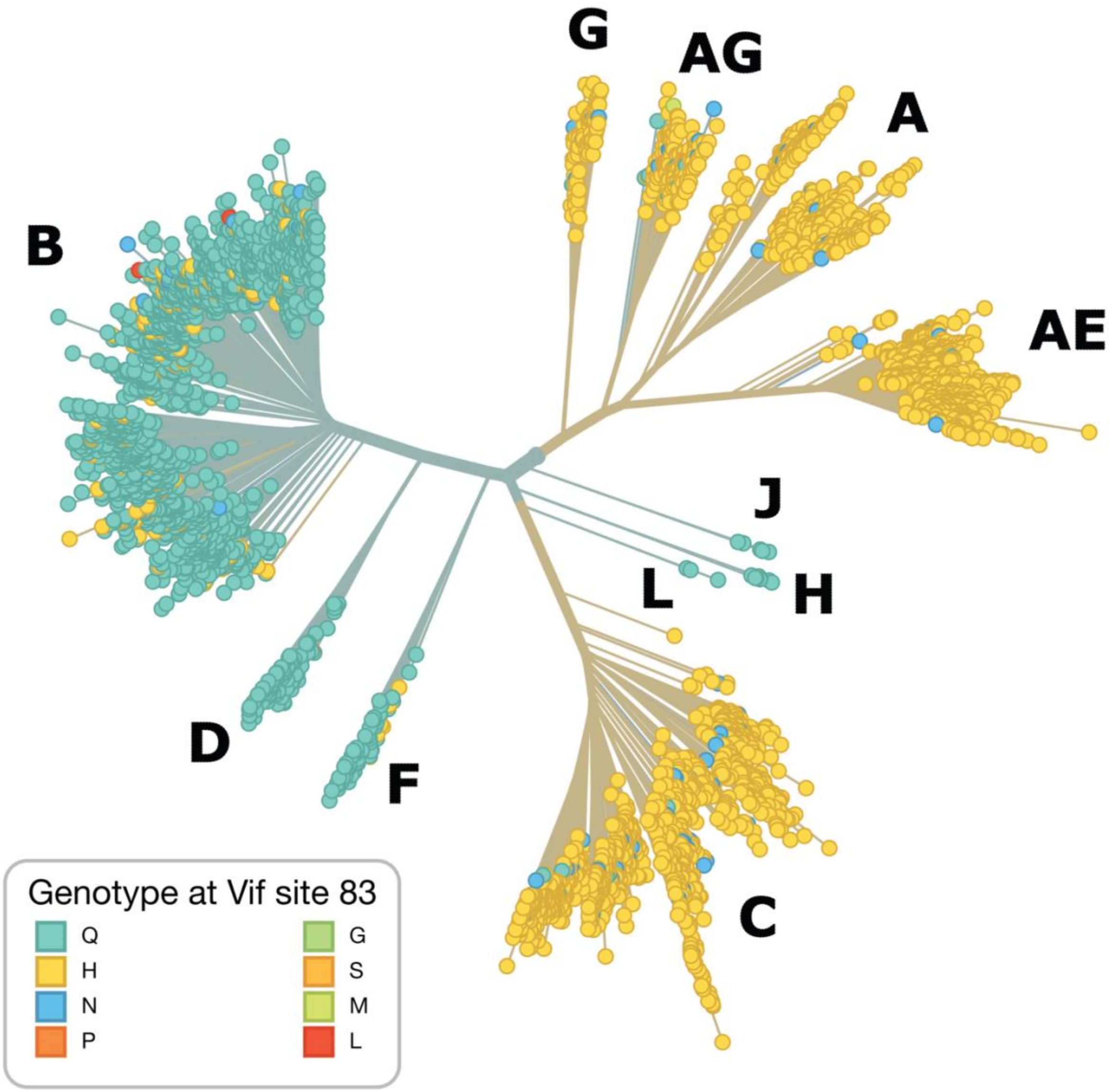

Supplementary Figure 2: Genotypic analysis of Vif position 83 across 3,310 HIV-1 genomes. Distribution of amino acid variants at Vif residue 83 based on global sequences in the HIV Sequence Database (https://hiv.lanl.gov/). Data were retrieved on October 15, 2025, from Nextstrain's LANL-HIV-DB view (https://nextstrain.org/groups/LANL-HIV-DB/HIV/genome). Clade designations are indicated by bold letters.

bioRxiv preprint doi: https://doi.org/10.1101/2025.10.20.683452; this version posted October 20, 2025. The copyright holder for this preprint (which was not certified by peer review) is the author/funder, who has granted bioRxiv a license to display the preprint in perpetuity. It is made available under aCC-BY 4.0 International license.

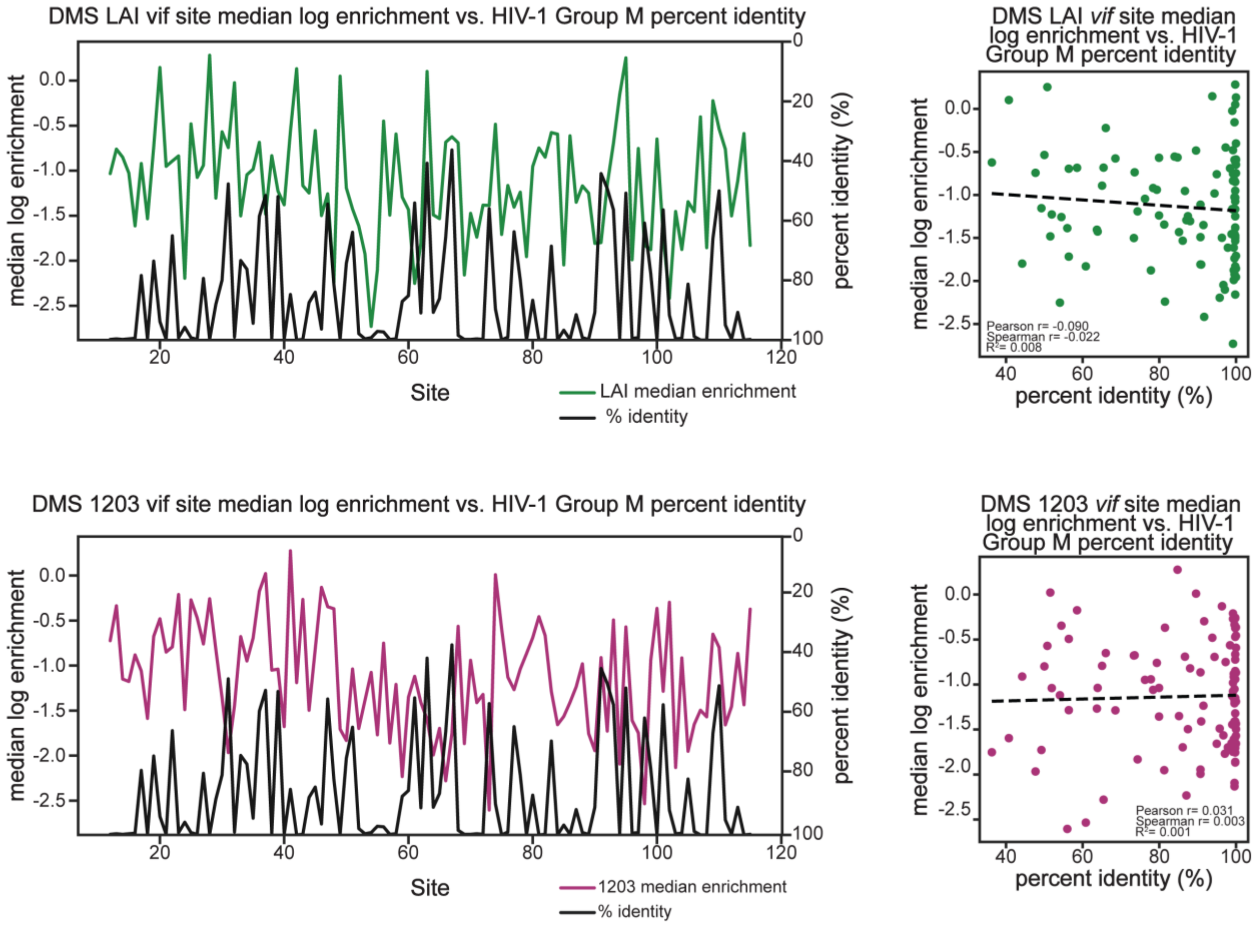

Supplementary Figure 3: Comparison of functional constraint (via DMS) and evolutionary conservation in HIV-1 Vif. Per-site median log enrichment scores from deep mutational scanning (DMS) of HIV-1 Vif (strain LAI, top row; strain 1203, bottom row) were compared against natural sequence conservation across HIV-1 Group M sequences. Left panels: Median log enrichment values (green for LAI, purple for 1203) are plotted residue by residue across Vif (sites 12–115) alongside percent identity relative to the modal (consensus) amino acid at each site in a Group M alignment (black line, right y-axis). Right panels: Scatterplots of per-site median enrichment versus consensus percent identity. Each dot corresponds to one Vif residue. Linear regression lines are shown (dashed black), along with correlation statistics (Pearson's r, Spearman's ρ). These analyses test whether functional constraint observed in the DMS assays corresponds to evolutionary conservation across natural HIV-1 sequences.

bioRxiv preprint doi: https://doi.org/10.1101/2025.10.20.683452; this version posted October 20, 2025. The copyright holder for this preprint (which was not certified by peer review) is the author/funder, who has granted bioRxiv a license to display the preprint in perpetuity. It is made available under aCC-BY 4.0 International license.

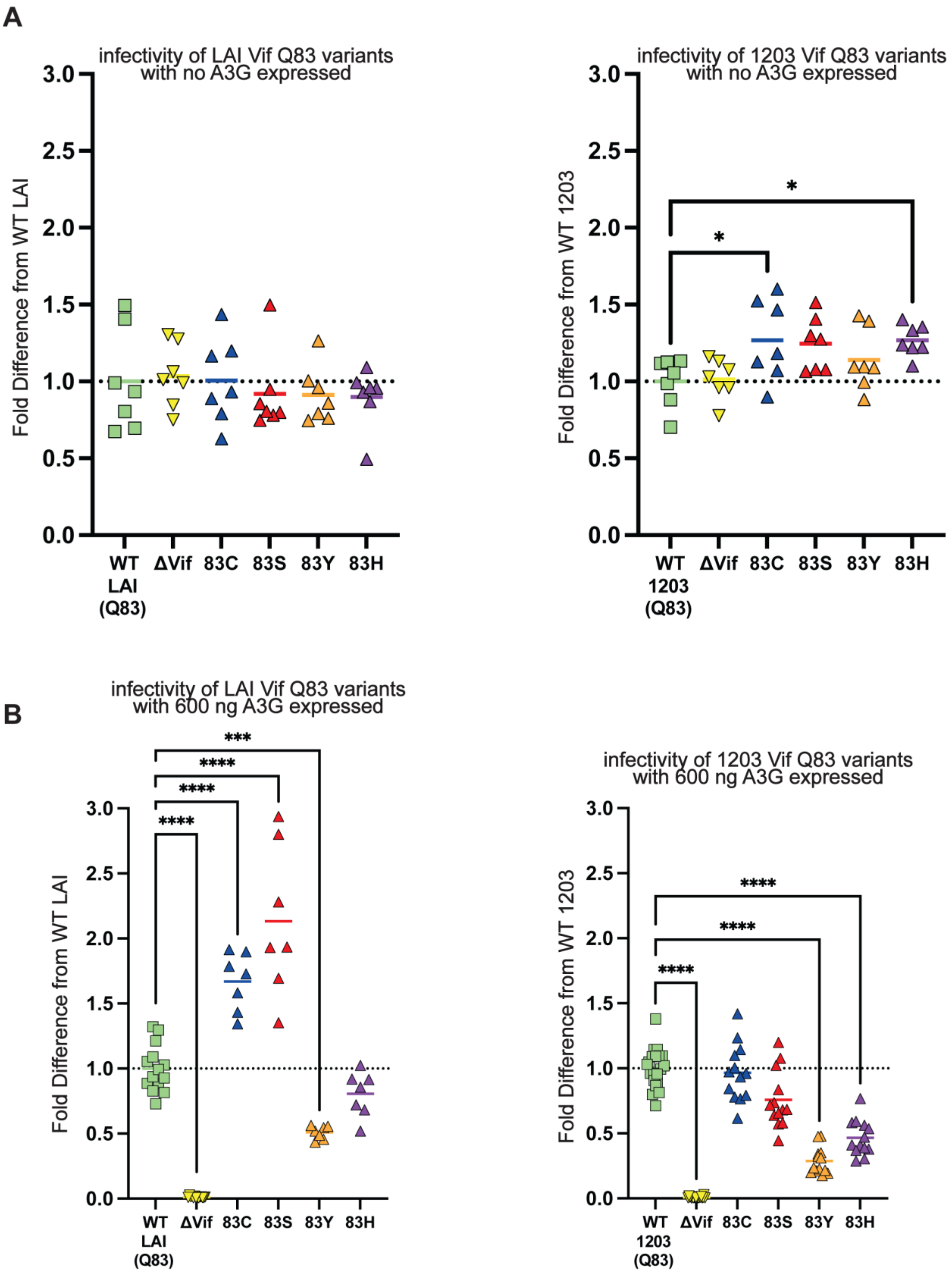

bioRxiv preprint doi: https://doi.org/10.1101/2025.10.20.683452; this version posted October 20, 2025. The copyright holder for this preprint (which was not certified by peer review) is the author/funder, who has granted bioRxiv a license to display the preprint in perpetuity. It is made available under aCC-BY 4.0 International license.

Supplementary Figure 4: Infectivity assays of Q83 Vif variants with and without A3G. A. Infectivity of replication-competent viruses encoding Q83 variants in the LAI/1203 Vif backgrounds when no A3G is expressed. Values are then expressed as fold change relative to wild-type. B Infectivity of replication-competent viruses encoding Q83 variants in the context of 600ng A3G, normalized within each experiment to a no-A3G control (panel A); values are then expressed as fold changed relative to wild-type. Wild-type and ΔVif viruses used as controls. Statistical significance was assessed using one-way ANOVA followed by Dunnett's multiple comparisons test, comparing each variant to wild-type Vif: ns = not significant ($P > 0.05$); *$P \leq 0.05$; **$P \leq 0.01$; ***$P \leq 0.001$; ****$P \leq 0.0001$.
